# Global Gene Expression Profiling of Human Osteosarcomas Reveals Metastasis-Associated Chemokine Pattern

**DOI:** 10.1155/2012/639038

**Published:** 2012-02-28

**Authors:** Heidi M. Namløs, Stine H. Kresse, Christoph R. Müller, Jørn Henriksen, Rita Holdhus, Gunnar Sæter, Øyvind S. Bruland, Bodil Bjerkehagen, Vidar M. Steen, Ola Myklebost

**Affiliations:** ^1^Department of Tumour Biology, The Norwegian Radium Hospital, Oslo University Hospital, 0310 Oslo, Norway; ^2^Cancer Center, Sorlandet Hospital Kristiansand, 4604 Kristiansand, Norway; ^3^Center for Medical Genetics and Molecular Medicine, Haukeland University Hospital, 5021 Bergen, Norway; ^4^Department of Oncology, The Norwegian Radium Hospital, Oslo University Hospital, 0310 Oslo, Norway; ^5^Department of Pathology, The Norwegian Radium Hospital, Oslo University Hospital, 0310 Oslo, Norway; ^6^Department of Clinical Medicine, University of Bergen, 5020 Bergen, Norway; ^7^Norwegian Microarray Consortium, Department of Molecular Biosciences, University of Oslo, 0310 Oslo, Norway

## Abstract

Global gene expression analysis was performed on a panel of 23 osteosarcoma samples of primary and metastatic origin using the Applied Biosystems Gene Expression Array System. When comparing the primary tumours with the metastases, we found a significantly increased expression of genes involved in immunological processes, for example coding for cytokines and chemokines, in the metastatic samples. In addition, a comparison of the gene expression in primary samples from patients with or without metastases demonstrated that patients who later developed metastases had high expression of the chemokine (C-X-C motif) receptor 4 (*CXCR4*), similar to the metastatic samples, suggesting that these signal molecules play an important role in promoting metastasis. Increased knowledge of mechanisms and interactions between specified molecular signalling pathways in osteosarcomas could lead to a more rational strategy for development of targeted therapy.

## 1. Introduction

Osteosarcoma is the most frequent primary malignant tumour of bone in children and adolescents, with a peak incidence at the age of 15–19 years, and has an annual incidence of 4/million/year world-wide (reviewed in [1, 2]). Osteosarcomas are rare, osteoid-producing malignant tumours that usually arise in the metaphyseal regions of long bones, in particular, the distal femur, proximal tibia, and proximal humerus [2]. Although most osteosarcomas are diagnosed without a predisposing condition, approximately 15% arise in adults secondary to a predisposing genetic condition (Li-Fraumeni syndrome, hereditary retinoblastoma; RB), disease (Paget disease of bone), or prior treatment (radiation) [2].

Most conventional osteosarcomas are high-grade tumours with a complex karyotype that displays numerous genetic aberrations [2]. Despite the advances in multimodal treatment combining adjuvant chemotherapy and surgical-wide resection, the 5-year survival rate for patients diagnosed with osteosarcoma without presence of metastasis remains in the order of 60–65% (reviewed in [3, 4]). Metastases are the leading cause of cancer-related death, and around 13–27% of the osteosarcoma patients have detectable metastases at diagnosis [3–6], whereas 40% will develop metastases at a later stage [4]. The metastatic process shows a tropism for lungs (80%), with skeleton as the second most common site [4]. The 5-year survival rate for osteosarcoma patients with primary metastases is in the range of 20–29% [4, 7, 8].

Molecular pathways contributing to osteosarcoma development and progression have recently been discovered (reviewed in [9, 10]), and this may facilitate better diagnosis and prognostication, as well as the development of new treatment strategies. The molecular alterations contributing to metastasis in osteosarcomas are increasingly being understood (reviewed in [4]), and several studies have employed microarray gene expression profiling to identify genes involved in the metastasis process. Comparisons of high- and low-metastatic osteosarcoma cell lines using microarrays have identified several differentially expressed genes related to growth arrest and apoptosis [11], as well as adherence, motility, and/or invasiveness [12]. Another study identified two genes not previously linked to osteosarcoma, epiregulin (*EREG*) and carbohydrate (N-acetylglucosamine-6-O) sulfotransferase 2 (*CHST2*), both predictive for survival [13]. A recent gene expression profiling of osteosarcoma patients who did and did not develop metastasis revealed a number of differentially expressed genes related to immunological functions, particularly macrophages [14].

Due to the high incidence of metastasis and low survival rate in metastatic osteosarcoma patients, we wanted to investigate the differences in gene expression pattern between primary and metastatic tumours. Microarray gene expression analysis was performed on a panel of 23 osteosarcoma samples of primary and metastatic origin, and the expression patterns were compared in order to identify differentially expressed genes and molecular signalling pathways involved.

## 2. Materials and Methods

### 2.1. Biological Material

Twenty-three human osteosarcomas were selected from a tumour panel at the Department of Tumour Biology at the Norwegian Radium Hospital, collected during two decades (1983–2004). Clinical samples were collected immediately after surgery, cut into small pieces, frozen in liquid nitrogen, and stored at −80°C until use. The informed consent used and the collection of samples were approved by the Ethical Committee of Southern Norway (Project S-06133).

All tumours were revised at the time of the study by the pathologist (BB) and diagnosed according to the current World Health Organization classification [2]. The clinical information was retrieved anonymously from the MEDinsight database at the Norwegian Radium Hospital (http://medinfo.net/medinsight/). The panel consisted of 12 primary tumours obtained from open biopsies or surgical specimens and 11 tumours obtained from surgical specimens of distant metastases. Of the 12 primary tumours, 7 were from patients who later developed metastases and 4 from those who did not (clinical information about the metastatic process was not available for one primary tumour). The follow-up of the patients who did not develop metastasis was in the range 5–19 years. Median patient age was 18 (range 11–50), and the gender ratio (female : male) was 1 : 1.3. Clinical information on the tumour samples are given in Table 1.

Two normal bone samples were included for the quantitative real-time RT-PCR experiments. Bone1 was collected from the femur of a renal cell carcinoma patient and Bone2 from the tibia of an osteosarcoma patient. For both patients, the normal samples were collected distant from the margin of the tumours.

### 2.2. Microarray Experiments

The microarray experiments were performed with the Applied Biosystems Gene Expression Array System, and the samples were hybridised on the Human Genome Survey Microarray V2.0 (Applied Biosystems, Foster City, CA, USA) with 32 k probes covering 29 k genes. The complete dataset can be viewed in the Gene Expression Omnibus (GEO) microarray database (http://www.ncbi.nlm.nih.gov/geo/, accession number GSE32981).

### 2.3. RNA Isolation and Hybridization

RNA was extracted by TRIzol (OS3, 4, 12, 18, 21, 41, 47, 48, 50, 51, 53, 55) or GTC (OS11, 14, 15, 17, 19, 20, 23, 25, 29, 30, and 32) using standard protocols. RNA purity and quantity were measured on a NanoDrop ND-1000 spectrophotometer (Nanodrop Technologies, Wilmington, DE, USA), and RNA integrity was evaluated on an Agilent 2100 Bioanalyzer (Agilent Technologies Inc., Santa Clara, CA, USA) using the total-RNA chip. The RNA was amplified, hybridised on the Human Genome Survey Microarray V2.0 (Applied Biosystems), and scanned according to the manufacturer's protocols.

### 2.4. Preprocessing and Filtering

The microarray image files were pre-processed with the AB1700 software (Applied Biosystems) and the resulting data files were stored in BASE [15]. The data were further processed with the R package ABarray, which is part of the Bioconductor project (http://www.r-project.org/) [16]. The data were quantile normalized, log_2_ transformed, and missing values were imputed using average values from the other arrays in the subgroup. The osteosarcoma samples were divided into two groups of primary or metastatic origin. Weakly expressed probes were filtered away by defining that a probe is only detected if it has a signal-to-noise ratio (SNR) ≥ 3 in at least 50% of the samples in either subgroup primary or metastatic.

### 2.5. Data Analysis

Using the database Panther [17, 18], a Wilcoxon Rank-Sum test was performed to identify enriched biological pathways using all of the genes that were expressed above the detection limit and mapped to Celera ID.

The data were imported to J-Express 2.7 [19], and the values were merged to gene level using the max probe function. The Statistical Analysis of Microarrays (SAM) plug-in [20] was used to identify differentially expressed genes between the primary and the metastatic samples, as well as between the primary samples that did and did not develop into metastases. Significant genes were identified using the *d*-value, which measures the strength of the relationship between gene expression and the response variable. An analysis was performed on the discriminatory gene lists where the genes were categorized based on enriched Gene Ontology (GO) processes using Panther. Hierarchical clustering was done in J-Express, and the gene expression is given relative to the mean expression level in all the samples.

### 2.6. Quantitative Real-Time RT-PCR

Quantitative real-time RT-PCR was performed using TaqMan Gene Expression Assays (Applied Biosystems, California, USA). The expression level of the gene chemokine (C-X-C motif) receptor 4 (*CXCR4*, assay ID Hs00237052_m1) was determined in 19 of the tumour samples and 2 normal bone samples. The genes beta-2-microglobulin (*B2M*, assay ID Hs99999907_m1), TATA-box binding protein (*TBP*, assay ID Hs99999910_m1), and eukaryotic 18S rRNA (*18S*, assay ID Hs99999901_s1) were used as endogenous controls for normalization. Universal Human Reference RNA (Stratagene, California, USA) was used as a reference.

The High Capacity cDNA Archive Kit (Life Technologies) was used to synthesize cDNA, and real-time PCR was performed using the ABI 7500 Real Time PCR System and software, essentially as described in the protocol supplied by the manufacturer (Applied Biosystems). Each assay included (in duplicate) a standard curve of six serial dilutions of the Universal Human Reference RNA cDNA (ranging from 100 ng to 100 pg), 10 ng of each tumour and normal bone cDNA, and a no-template control. The expression levels were determined from the standard curves as described by the manufacturer. The expression level of *CXCR4* was normalized with the average expression of the three endogenous controls, and the relative expression of the tumour samples was compared to the average expression of the two normal bone samples.

## 3. Results 

### 3.1. Comparison of Primary and Metastatic Tumours

Gene expression profiling was performed on a panel of 23 human osteosarcoma samples of primary and metastatic origin. The clinical information on the tumour samples is given in Table 1. Twelve primary and 11 metastatic samples were analysed to identify differences in gene expression and pathways with enrichment of differentially expressed genes between the two groups. After SNR filtering, 22,510 genes were expressed above the detection limit, of which 21,378 had Celera IDs. Unsupervised hierarchical clustering of all the samples revealed no specific patterns, and the primary and metastatic samples clustered intermingled (data not shown). The analysis using Panther identified several pathways with enriched differential expression between the primary and metastatic samples, and these are listed in Table 2. The genes in the pathway were generally higher expressed in the metastases than in the primary samples. The most prominent pathways are involved in immunological processes and chemokine and cytokine signalling, as well as pathways like the EGF receptor signalling pathway, including genes coding for the FBJ murine osteosarcoma viral oncogene homolog (*FOS*), early growth response 1 and -2 (*EGR1* and -*2*).

**Figure 1:**
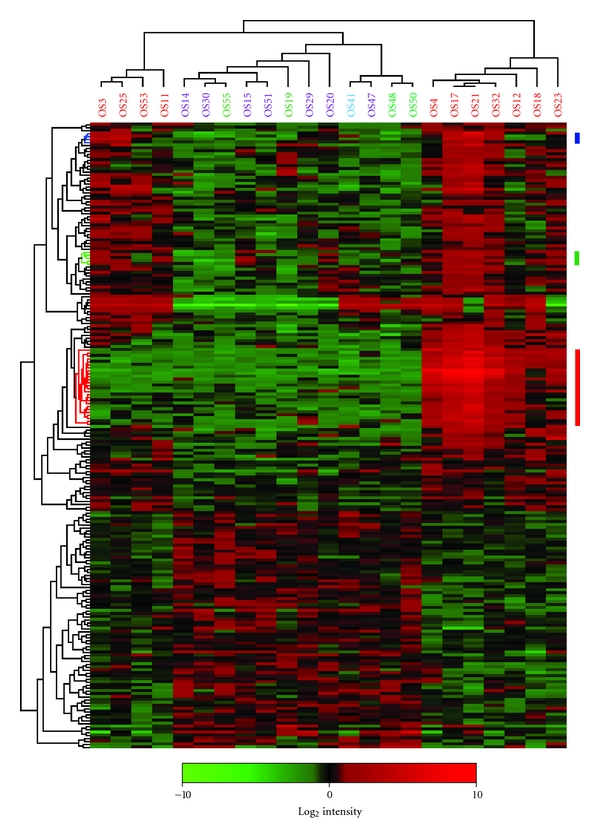
Hierarchical clustering of all tumours based on the top-210 significant genes differentially expressed between metastases and primary tumours, identified by SAM analysis. Samples coloured in red, metastases; lilac, primary samples from patients who later developed metastases; green, primary samples from patients who did not develop metastases blue, clinical information on metastases not available. Gene cluster coloured in blue, *CXCL1, -2*, -*3*, *IL6,* and *LOC131873*; green, *EGR1*, -*2*, *FOSB*, *FOS,* and *JUNB*; red, surfactant genes. Red, increased gene expression; green, decreased gene expression. The cluster was made using average linkage and Pearson's correlation.

The primary and metastatic samples were further compared by SAM analysis in order to identify the most differentially expressed genes separating these two groups, and the top-210 gene list based on the *d*-value is given in Supplementary Table 1 available at doi:10.1155/2012/639038. The majority of these genes were upregulated in the metastases compared to the primary tumours.  Figure 1 shows the hierarchical clustering of all the tumours based on the expression pattern of these 210 genes (the same figure with the gene names indicated is given in Supplementary Figure 1). The samples were separated into two main clusters, one consisting of all primary and a subset of the metastatic samples and one with only metastatic samples. Among the primary samples, there was one subcluster that strongly diverged from the other samples, consisting of three samples that did not metastasize and the one sample with unknown metastatic status. Among the genes separating the metastatic samples into different subclusters, a high number of surfactants were present, being highly expressed in all the samples of the subcluster that only contained lung metastases. In addition, several interesting clusters of genes were observed to be upregulated in both of the metastatic subclusters, like the group consisting of chemokine (C-X-C motif) ligand 1, -2, and -3 (*CXCL1*, -*2*, and -*3*) and interleukin 6 (interferon, beta 2) (*IL6*), as well as the group consisting of *EGR1*, -*2*, *FOSB*, *FOS,* and jun B proto-oncogene (*JUNB*), both highlighted (blue and green colour, resp.) in Figure 1.

To further explore the function of these 210 differentially expressed genes, they were classified into Gene Ontology (GO) Biological process and Molecular function, listed in Table 3. Interestingly, there was a significant overrepresentation of genes involved in immunity and defence, especially cytokines and chemokines like *CXCL1*, -*2*, -*3*, -*5*, *IL1B*, -*6*, -*8*, -*17D* and oncostatin M (*OSM*). These signal molecules have a higher expression in metastatic samples compared with primary samples, and the high expression level seems to be strongly associated with metastasis. In addition, a group of surfactants, surfactant protein A1, -A2, -B, -C, and -D (*SFTPA1*, -*A2*, -*B*, -*C,* and -*D*) was observed to be higher expressed in a subset of the metastatic samples, contributing to a gene cluster that distinguished between lung and non-lung metastases (coloured red in Figure 1).

### 3.2. Comparison of Primary Tumours with Different Capability to Metastasize

**Figure 2:**
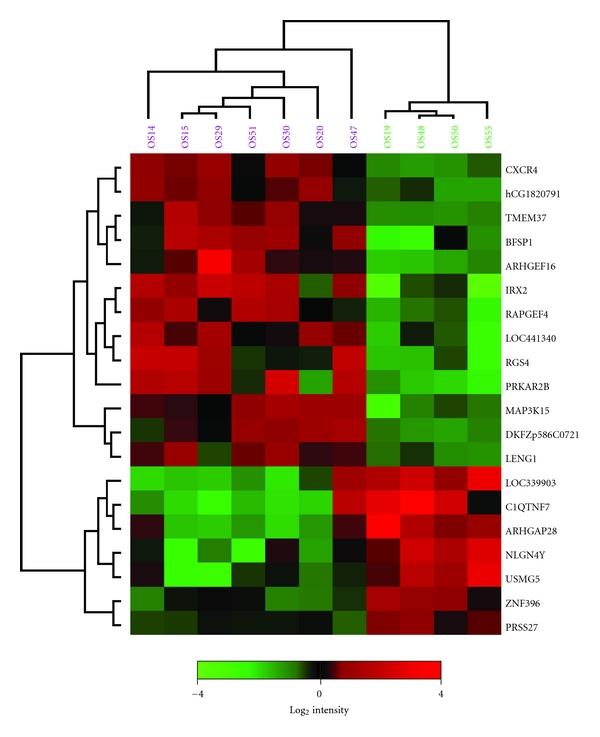
Hierarchical clustering of all primary tumours based on the top-20 significant genes differentially expressed between primary samples from patients who developed metastases and those who did not, identified by SAM analysis (one primary tumour with no clinical information on metastases was excluded). Samples coloured in lilac, primary samples from patients who later developed metastases; green, primary samples from patients who did not develop metastases. Red, increased gene expression; green, decreased gene expression. The cluster was made using average linkage and Pearson's correlation.

To further look into the genes apparently involved in the metastatic process, the primary samples from the patients who developed metastases were compared with those who did not. A SAM analysis of seven primary samples that metastasized and four that did not resulted in a short list of genes that were differentially expressed between the two groups of primary samples. The top-20 gene list based on the *d*-value is shown in Table 4.  Figure 2 shows the hierarchical clustering of the primary samples based on the expression of these 20 genes. The primary samples that developed into metastases clustered separately from the primary samples that did not. Thirteen of the differentially expressed genes were upregulated in the primary samples that developed into metastases, whereas seven genes were downregulated. Among the genes that were upregulated in the primary samples from patients who later developed metastases was the chemokine (C-X-C motif) receptor 4 (*CXCR4*), known to be involved in the metastatic process.

**Figure 3:**
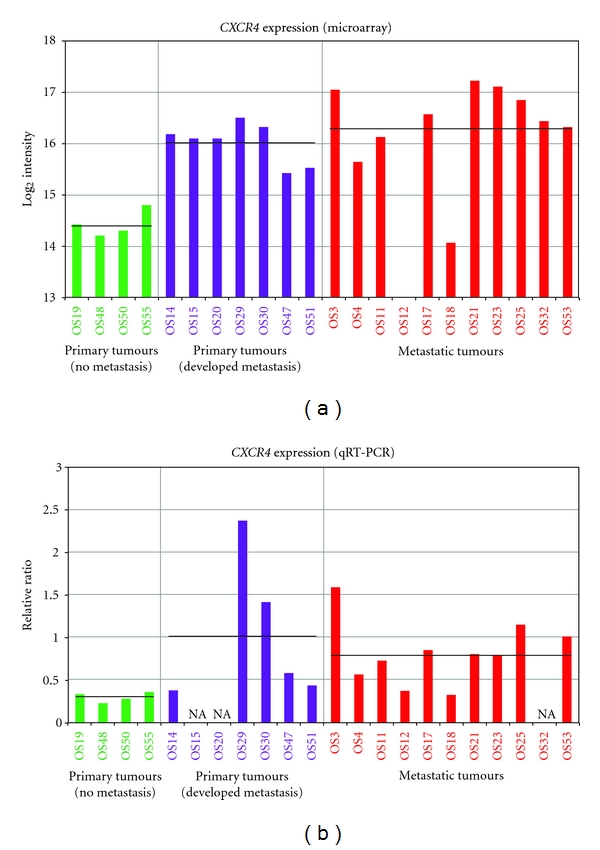
Relative *CXCR4* expression in primary tumours from patients who did or did not develop metastases and in metastatic samples (one primary tumour with no clinical information on metastases was excluded) based on (a) microarray data and (b) quantitative real-time RT-PCR data. For the quantitative real-time RT-PCR data, the expression levels have been normalised to the average expression of three housekeeping genes (*18S*, *B2M* and *TBP*) and compared to the average expression of two normal bone samples. The average expression level in each sample group is indicated with a black bar. NA, not available.

Figure 3(a) shows the expression level of the *CXCR4* gene in all the samples. *CXCR4* was expressed at the same level in metastatic samples and in primary samples that developed metastases, with a significantly lower expression level in primary samples that did not metastasize compared to the two other groups (*P* < 0.001 for both comparisons). The expression was also significantly different between all the primary samples combined and the metastatic samples (*P* < 0.05). The expression level was also determined in 19 of the tumour samples compared to two normal bone samples using quantitative real-time RT-PCR, and this is shown in Figure 3(b). The relative expression levels were in general similar using the two methods, with higher expression of *CXCR4* in the primary samples that developed metastases and the metastatic samples. Most samples showed similar expression levels, only OS14 was markedly different. However, there were smaller relative differences in expression level between the samples based on the quantitative real-time RT-PCR data, and the expression was not significantly different between the groups here.

## 4. Discussion

Osteosarcomas show complex genomic changes with few, if any, consistent chromosomal aberrations, which makes it difficult to identify the molecular features that underlie the development of this type of cancer. The aim of this study was to investigate the differences in gene expression pattern between primary and metastatic tumours, and a number of pathways and genes with differential expression were identified (Tables 2 and 3 and Figure 1). In general, the genes were more highly expressed in the metastases than in the primary osteosarcoma tumour samples. Of particular interest, we identified a number of immunological processes, including the T cell and B cell activation, as well as chemokine and cytokine signalling pathways. Chemokines are chemotactic cytokines, a family of small cytokines or proteins secreted by cells, and the major role of chemokines is to control and direct the migration of cells. It has been increasingly known that chemokines play an important part in regulation of the metastatic cascade in a wide range of tumours, including osteosarcomas [21–24]. Tumour cells that are attracted by chemokines have an increased expression of particular chemokine receptors on their surface. The cells migrate towards a signal of increasing chemokine concentration provided by the source of the chemokine, and this process enables them to migrate to secondary tissues where chemokine ligands are highly expressed. Part of the identified differences between primary and metastatic samples could be due to the origin of the samples, although the histology of primary and pulmonary metastatic osteosarcoma samples has been reported to be similar in about 60% of the cases [25, 26]. 

The chemokines *CXCL1*, -*2*, -*3*, -*5,* and *IL8* (*CXCL8*) were among the top-210 genes that were observed to be upregulated in metastases compared with primary tumours, and they showed highly correlated expression patterns (Figure 1, Supplementary Table 1, and Supplementary Figure 1). This is similar to previous observations in breast cancer tumours and cell lines, where these chemokines have been suggested to account for a higher aggressiveness of ER*α*-positive tumours [27]. These genes are located in the 4q21 region, and although the gene cluster was not amplified in breast cancer, it was observed to be coregulated at the transcriptional level. Regions in 4q have been shown to have increased copy number in osteosarcomas [28, 29], but amplification of the 4q21 region has not recurrently been reported. In breast cancer, *CXCL8* expression level strongly correlated with activating transcription factor 3 (*ATF3*), c-Jun and JunB, members of the AP-1 transcription factor complex [27], and *ATF3* and *JunB* were also observed to be upregulated in the metastatic osteosarcoma samples investigated here.

Several other chemokines were upregulated in the metastatic osteosarcomas, including *OSM*, which has been claimed to have both pro-inflammatory and anti-inflammatory effects. OSM has been shown to induce bone loss and sensitized rat osteosarcoma to apoptosis [30] and induce differentiation of chondrosarcoma and osteosarcoma cells [31, 32]. However, several studies show that OSM may enhance tumour progression and metastasis. Stimulation of human and canine osteosarcoma cells by OSM has been shown to promote invasive behaviour through activation of signal transducer and activator of transcription 3 (acute-phase response factor, STAT3) [33], and OSM treatment increased migration and enhanced epithelial-to-mesenchymal transition in several tumours [34–36].

The hierarchical clustering based on the top-210 differentially expressed genes separated the metastatic samples into two subclusters (Figure 1). The smaller metastatic cluster, which was more similar to the primary samples, consisted of one pulmonary, one skeletal, and two samples that formed multiple metastases of both the lungs and skeleton. The major cluster of metastatic samples only included pulmonary metastases, and these samples showed high expression of surfactants. SP-A, SP-B, SP-C, and SP-D are expressed by type II alveolar epithelia or Clara cells, assumed to be progenitors of pulmonary adenocarcinomas, and have been used as markers of metastatic and micrometastatic pulmonary adenocarcinoma and nonsmall cell lung carcinomas [37]. It seems likely that the detection of these surfactants may be due to contamination of lung tissue in the tumour samples, although it is also possible that the lung microenvironment induces these genes in the osteosarcoma cancer cells.

Interestingly, in the comparison of primary tumours with different capability to metastasize, primary samples that did not metastasize showed significantly lower expression of the chemokine receptor *CXCR4* than primary samples from patients who later developed metastases (*P* < 0.001) (Table 4 and Figures 2 and 3(a)). The expression level was similar between the primary samples that metastasized and the metastatic samples. Although *CXCR4* was not among the top-ranked genes from the SAM analysis of primary tumours and metastases, the expression level was significantly different between all the primary tumours and the metastases (*P* < 0.05), most likely due to the lower level of expression in the primary samples that did not metastasize. The expression level was also confirmed using quantitative real-time RT-PCR (Figure 3(b)), and although the patterns in general were similar between the two methods, no significant differences in expression were observed between the groups based on this data.

Two of the metastatic samples, OS11 and OS18, originated from patients that did not develop metastases until 15 and 10 years after diagnosis, respectively. OS11 is a parosteal osteosarcoma but expressed *CXCR4* at the same level as the other metastatic samples, while OS18 showed a low level of *CXCR4* expression. Both samples were untreated, in contrast to the other metastatic samples, but the clustering analyses showed that these two cases were not different from the other metastatic samples, justifying the inclusion of these samples in the study. Similarly, two primary samples were treated (OS14 and OS19), in contrast to the other primary samples, but these were also not different from the other primary samples. However, OS14 was the only samples showing markedly different expression level of *CXCR4* depending on the method used.

This study is the first to use expression profiling to identify that differential expression of *CXCR4* separates primary samples with different capabilities to metastasize. Although *CXCR4* was not among the identified genes in a recent publication by Buddingh et al. [14], the significance of CXCR4 in metastasis development in osteosarcoma and other bone cancers has previously been reported. CXCR4 is a specific receptor for the ligand SDF-1 (stromal-derived-factor-1, also called CXCL12), and the CXCR4/CXCL12 axis has been shown to be important for tumour progression in a high number of cancer types [38]. In a mouse model, the tumour cells with CXCR4 receptor were chemoattracted by CXCL12, migrated through the lymphatic and vascular system, and arrested in CXCL12 rich organs like the bone and lungs [39]. Laverdiere et al. [24] observed no significant difference between *CXCR4* expression in primary and metastatic samples, but the level of *CXCR4* expression was inversely correlated with the presence of metastases at diagnosis and survival (event-free survival and metastatic-free survival), as patients with tumors expressing *CXCR4* had a worse survival. However, in a study by Oda et al. [40], higher immunohistochemical CXCR4 expression was observed in metastases compared with primary tumours. Contradicting the above observation, a higher frequency of canine osteosarcoma primary tumours than pulmonary metastases expressed CXCR4 protein [41]. In an analysis of Ewing sarcoma, another bone cancer, *CXCR4* correlated with metastases, and *CXCR4* in combination with *CXCR7* were shown to be prognostic indicators for patient survival [23]. In chondrosarcoma of bone, CXCR4 showed higher immunohistochemical staining in high-grade than in low-grade samples, being a potential marker of aggressiveness [42].

A note to make is that the RNA samples used in this study were isolated using two different methods, which may influence the gene expression detected. The type of RNA isolation was randomly distributed between the groups of primary and metastatic samples, and with regard to other clinical properties (Table 1). Although it cannot be ruled out that the RNA isolation method has had an effect on the detected expression levels of *CXCR4*, the increasing evidence of a role of CXCR4 in osteosarcoma metastasis makes us believe that the expression differences observed here is due to the sample types and not the RNA isolation methods.

Osteosarcoma consists of both tumour cells derived from the mesenchyme as well as infiltrating mononuclear cells [43], hence chemokines could be expressed both by tumour cells as well as by the stroma cells in their proximity and at metastatic sites. Preliminary microarray results showed that *CXCR4* and interesting chemokine ligands like *CXCL1*, -*2*, -*3,* and -*5* are only highly expressed in clinical samples and not in xenografts or cell lines (Namløs et al., unpublished data), a finding supported by a previous study of osteosarcoma patient samples and cell lines [24]. This suggests that it is infiltrating stroma (macrophages) and not the tumour cells that is the major source of chemokine expression in osteosarcoma, or possibly that only the human macrophages are able to induce the expression of chemokines in the tumour cells. However, Perissinotto et al. [39] detected the CXCR4 receptor and a functional CXCR4/CXCL12 axis in non-confluent osteosarcoma cell lines. To further investigate this, immunohistochemical staining should be performed to examine the CXCR4 protein expression in the different cell types, which has previously been performed for other chemokine receptors in osteosarcoma [44].

Inhibition of the CXCR4/CXCL12 pathway by a peptide CXCR4 antagonist reduced the development of osteosarcoma murine lung metastases [45]. *In vivo* studies on breast cancer demonstrated that treatment with miRNA or antibodies against CXCR4 impaired migration and the development of murine lung metastases [21, 46]. This indicates that small molecule antagonists against CXCR4 can interfere with tumour progression and metastasis and may potentially be used for new therapeutic inventions.

## 5. Conclusion 

Although the number of samples analysed was moderate, our results add to the increasing number of studies linking CXCR4 to metastasis, suggesting that the expression level of *CXCR4* may possibly be used as a prognostic factor in osteosarcoma. The identification of chemokine pathways that may promote cancer spread could give clinically useful biomarkers for the prediction of particularly aggressive tumours and might suggest therapeutic regimens that may target such tumours.

## Supplementary Material

Supplementary Table 1: Top-210 genes differentially expressed between primary tumours that did and did not develop into metastasis by SAM analysis.
Supplementary Figure 1: Unsupervised hierarchical clustering of all tumours based on the top-210 significant genes differentially expressed between metastases and primary tumours, identified by SAM analysis.

## Figures and Tables

**Table 1:** Clinical information on the osteosarcoma samples.

Sample	Sample origin	Patient age/sex	Subtype	Grade^1^	Primary location	Metastasis location	Chemotherapy^2^	Response^3^	Treated sample	Metastasis (months)^4^	Follow-up (months)^5^	Status
OS3	Met	17/M	Obl/Cho	4	Femur	Lung	Yes (SSG II + VI)	NA	Yes	23	45	DD
OS4	Met	15/M	Obl	4	Femur	Lung	Yes (SSG II)	Poor	Yes	14	37	DD
OS11	Met	22/M	Par	4	Femur	Multiple^6^	No	—	No	185	193	DD
OS12	Met	41/M	Obl/Cho	4	Femur	Lung	Yes (SSG VI + VIII)	NA	Yes	9	49	DD
OS17	Met	18/M	Obl + Cho	4	Humerus	Lung	Yes (SSG II + VI)	NA	Yes	25	49	DD
OS18	Met	26/M	Obl	3	Fibula	Lung	No	—	No	117	353	DD
OS21	Met	17/F	Obl	4	Femur	Lung	Yes (SSG VIII)	Poor	Yes	MD	33	DD
OS23	Met	11/F	Obl	4	Femur	Lung	Yes (SSG VIII)	NA	Yes	MD	229	NED
OS25	Met	19/M	Fbl	4	Fibula	Skeleton	Yes (SSG VI + VIII)	NA	Yes	MD	44	DD
OS32	Met	24/M	Fbl	4	Tibia	Lung	Yes (SSG VI)	Poor	Yes	6	208	NED
OS53	Met	18/M	Obl/Fbl	4	Humerus	Multiple^7^	Yes (ISG/SSG I)	Poor	Yes	26	68	DD
OS14	Prim	14/F	Obl/Fbl	4	Femur	Lung	Yes (SSG II)	Poor	Yes	MD	13	DD
OS15	Prim	49/F	Obl	4	Costa	Lung	Yes (NA)	NA	No	MD	3	DD
OS19	Prim	16/F	Obl	4	Tibia	—	Yes (SSG VIII)	NA	Yes	—	245	NED
OS20	Prim	17/F	Obl/Fbl	4	Humerus	Soft tissue	Yes (SSG VIII)	NA	No	139	151	DD
OS29	Prim	27/F	Obl	4	Pelvis	Lung	No	—	No	MD	4	DD
OS30	Prim	21/F	Obl	4	Femur	Lung	Yes (SSG VIII)	NA	No	MD	162	NED
OS41	Prim	11/M	Obl	4	Tibia	NA	No	—	No	NA	NA	NA
OS47	Prim	12/M	SC/Pleo	4	Humerus	Lung	Yes (SSG XIV + EURAMOS I)	Good	No	92	124	NED
OS48	Prim	50/M	Obl/Fbl	4	Tibia	—	Yes (SSG XIV)	NA	No	—	74	NED
OS50	Prim	11/M	Cho	4	Femur	—	Yes (SSG XIV)	Poor	No	—	108	NED
OS51	Prim	18/F	Obl/Tel	4	Femur	Lung	Yes (ISG/SSG II)	NA	No	MD	22	DD
OS55	Prim	17/F	Obl	4	Femur	—	Yes (SSG XIV)	NA	No	—	91	NED

Abbreviations: OS, osteosarcoma; Met, metastasis; Prim, primary tumour; M, male; F, female; Obl, osteoblastic; Cho, chondroblastic; Par, parosteal; Fbl, fibroblastic; SC, spindle cell; Pleo, pleomorphic; Tel, telangiectatic; NA, not available; DD, dead of disease; NED, no evidence of disease.

^1^Grading is based on a four-tiered system used in the Scandinavian Sarcoma Group (SSG).

^2^Chemotherapy has been given according to the indicated Scandinavian Sarcoma Group (SSG) protocols, Italian Sarcoma Group/Scandinavian Sarcoma Group (ISG/SSG) protocol or European, and American Osteosarcoma Study Group (EURAMOS) protocol (for more information, see http://www.ssg-org.net/index.htm).

^3^Histological evaluation.

^4^Time to first metastasis from diagnosis.

^5^Time to last follow-up from diagnosis.

^6^Multiple locations, lung, and lymph node.

^7^Multiple locations, lung, and skeleton.

**Table 2:** Enriched Panther pathways in the metastases compared to the primary tumours. The five most significant pathways are shown with the number of genes in the pathways. + or − signs indicate that for the genes belonging to this pathway, the distribution of fold change values is shifted towards higher or lower values, respectively, than the overall distribution of all genes that were uploaded. *P*-values were calculated from the Wilcoxon Rank-Sum test and Bonferroni corrected for multiple testing.

Pathways	# Genes	+/−	*P*-value
T cell activation	134	+	5.5E–14
Inflammation mediated by chemokine and cytokine signaling pathway	299	+	2.1E–11
B cell activation	92	+	7.2E–7
EGF receptor signaling pathway	175	+	1.0E–3
Integrin signaling pathway	244	+	1.8E–3

**Table 3:** GO groups with significant enrichment, based on a comparison of the top-210 significant genes differentially expressed between metastases and primary tumours against all genes present on the microarray. The number of genes in the total list and observed and expected number of genes in the gene list that map to the GO group are shown. + or − signs indicate over- or underrepresentation, respectively, of this GO group. *P*-values were Bonferroni corrected for multiple testing, *P*-value < 0.05.

GO group	# Genes total	Gene list			
		# Observed	# Expected	+/−	*P*-value
*Biological process*					
Immunity and defence	1365	30	11.3	+	3.7E–5
Granulocyte mediated immunity	59	6	0.5	+	1.7E–3
Macrophage mediated immunity	126	8	1.1	+	1.8E–3
Cell proliferation and differentiation	944	20	7.8	+	4.2E–3
Blood circulation and gas exchange	82	5	0.7	+	2.0E–2
Cell cycle control	390	12	3.2	+	1.7E–2
JNK cascade	60	5	0.5	+	3.2E–2

*Molecular function*					
Surfactant	9	5	0.7	+	2.8E–6
Chemokine	45	5	0.4	+	6.8E–3
Interleukin	34	4	0.3	+	4.1E–2

**Table 4:** Top-20 genes identified by SAM analysis as differentially expressed in the comparison of primary samples from patients who developed metastases and those who did not.

Gene symbol	Gene name	Fold change	*d*-value
CXCR4	Chemokine (C-X-C motif) receptor 4	3.1	4.3
LOC339903	—	−8.6	−3.7
TMEM37	Transmembrane protein 37	3.1	3.6
MAP3K15	Mitogen-activated protein kinase kinase kinase 15	4.1	3.4
BFSP1	Beaded filament structural protein 1, filensin	5.1	3.4
DKFZp586C0721	—	3.2	3.4
hCG1820791	—	2.7	3.3
IRX2	Iroquois homeobox protein 2	9.2	3.3
NLGN4Y	Neuroligin 4, Y-linked	−6.9	−3.3
C1QTNF7	C1q and tumor necrosis factor related protein 7	−12.5	−3.2
ARHGAP28	Rho GTPase activating protein 28	−6.7	−3.2
PRKAR2B	Protein kinase, cAMP-dependent, regulatory, type II, beta	6.8	3.2
RAPGEF4	Rap guanine nucleotide exchange factor (GEF) 4	4.1	3.2
LOC441340	—	4.1	3.2
RGS4	Regulator of G-protein signalling 4	5.8	3.1
ZNF396	Zinc finger protein 396	−2.4	−3.1
PRSS27	Protease, serine 27	−1.8	−3.1
USMG5	Upregulated during skeletal muscle growth 5	−5.9	−3.0
LENG1	Leukocyte receptor cluster (LRC) member 1	2.5	3.0
ARHGEF16	Rho guanine exchange factor (GEF) 16	4.9	3.0
