# Supplementary material for: Global Gene Expression Profiling of Human Osteosarcomas Reveals Metastasis-Associated Chemokine Pattern
Source: Sarcoma. 2012 Feb 28;2012:639038. doi: 10.1155/2012/639038 (PMC3324929; doi:10.1155/2012/639038)

**Supplementary Table 1.** Top-210 genes differentially expressed between primary tumours that did and did not develop into metastasis by SAM analysis (Namløs et al.)

| Number | Gene_Symbol | Gene_Name | Primary_Gene_ID | Entrez Gene ID | Celera Gene ID | UniGene ID | d[i] | de[i] | Delta[i] | FSN[i] | FDR[i] | Fold Change | q-val[i] |
|---|---|---|---|---|---|---|---|---|---|---|---|---|---|
| 1 | LOC284454 | null | 284454 | 284454 | hCG2001068 | Hs.436426 | 4.918 | 3.649 | 1.269 | 0 | 0 | 3.528 | 0 |
| 2 | CXCL2 | chemokine (C-X-C motif) ligand 2 | 2920 | 2920 | hCG16361.2 | Hs.75765 | 4.559 | 3.376 | 1.183 | 0 | 0 | 13.114 | 0 |
| 3 | PTGS2 | prostaglandin-endoperoxide synthase 2 (prostaglandin G/H synthase and cyclooxygenase) | 5743 | 5743 | hCG39885.3 | Hs.196384 | 4.337 | 3.234 | 1.103 | 0 | 0 | 7.841 | 0 |
| 4 | FOXF1 | forkhead box F1 | 2294 | 2294 | hCG17050.2 | Hs.155591 | 4.155 | 3.142 | 1.014 | 0 | 0 | 9.416 | 0 |
| 5 | RNMTL1 | RNA methyltransferase like 1 | 55178 | 55178 | hCG32500.2 | Hs.182729 | -4.119 | -3.647 | 0.472 | 0 | 0 | -1.969 | 0 |
| 6 | SLC2A3 | solute carrier family 2 (facilitated glucose transporter), member 3 | 6515 | 6515 | hCG22218.2 | Hs.419240 | 4.074 | 3.067 | 1.007 | 0 | 0 | 3.912 | 0 |
| 7 | SLC2A14 | solute carrier family 2 (facilitated glucose transporter), member 14 | 144195 | 144195 | hCG1730826.1 | Hs.210227 | 4.07 | 3.015 | 1.055 | 0 | 0 | 2.681 | 0 |
| 8 | SCGB1A1 | secretoglobin, family 1A, member 1 (uteroglobin) | 7356 | 7356 | hCG39843.2 | Hs.523732 | 3.999 | 2.965 | 1.035 | 0 | 0 | 11.506 | 0 |
| 9 | KRT19 | keratin 19 | 3880 | 3880 | hCG2043341 | Hs.514167 | 3.976 | 2.921 | 1.056 | 0 | 0 | 5.473 | 0 |
| 10 | IL8 | interleukin 8 | 3576 | 3576 | hCG16372.3 | Hs.624 | 3.9 | 2.884 | 1.016 | 0 | 0 | 6.508 | 0 |
| 11 | HOP | null | 84525 | 84525 | null | Hs.121443 | 3.857 | 2.852 | 1.005 | 0 | 0 | 6.389 | 0 |
| 12 | SUSD4 | sushi domain containing 4 | 55061 | 55061 | hCG23752.3 | Hs.497841 | -3.778 | -3.394 | 0.384 | 0.914 | 7.616 | -4.155 | 6.093 |
| 13 | NR4A1 | nuclear receptor subfamily 4, group A, member 1 | 3164 | 3164 | hCG37966.3 | Hs.524430 | 3.769 | 2.824 | 0.945 | 0.914 | 7.03 | 4.888 | 4.81 |
| 14 | EGR1 | early growth response 1 | 1958 | 1958 | hCG18777.1 | Hs.326035 | 3.748 | 2.797 | 0.95 | 0.914 | 6.528 | 4.201 | 4.81 |
| 15 | PRDM5 | PR domain containing 5 | 11107 | 11107 | hCG38692.3 | Hs.132593 | -3.739 | -3.239 | 0.499 | 0.914 | 6.093 | -2.266 | 6.093 |
| 16 | SERPINA1 | serpin peptidase inhibitor, clade A (alpha-1 antiproteinase, antitrypsin), member 1 | 5265 | 5265 | hCG2029168.1 | Hs.525557 | 3.705 | 2.772 | 0.933 | 0.914 | 5.712 | 6.586 | 4.81 |
| 17 | C4BPA | complement component 4 binding protein, alpha | 722 | 722 | hCG1986997 | Hs.1012 | 3.664 | 2.748 | 0.916 | 0.914 | 5.376 | 13.41 | 4.81 |
| 18 | SFTPD | surfactant, pulmonary-associated protein D | 6441 | 6441 | hCG1993456 | Hs.253495 | 3.601 | 2.729 | 0.872 | 0.914 | 5.077 | 17.991 | 4.81 |
| 19 | EGR2 | early growth response 2 (Krox-20 homolog, Drosophila) | 1959 | 1959 | hCG40234.2 | Hs.1395 | 3.59 | 2.709 | 0.882 | 0.914 | 4.81 | 4.577 | 4.81 |
| 20 | LOC253970 | null | 253970 | 253970 | hCG2013488 | Hs.509165 | 3.531 | 2.691 | 0.84 | 1.828 | 9.139 | 12.973 | 7.311 |
| 21 | NR4A2 | nuclear receptor subfamily 4, group A, member 2 | 4929 | 4929 | hCG38357.2 | Hs.165258 | 3.513 | 2.674 | 0.84 | 1.828 | 8.704 | 5.224 | 7.311 |
| 22 | null | null | hCG1995976 | null | hCG1995976 | Hs.313856 | -3.498 | -3.137 | 0.36 | 1.828 | 8.308 | -1.91 | 7.616 |
| 23 | SFTPC | surfactant, pulmonary-associated protein C | 6440 | 6440 | hCG23794.3 | Hs.1074 | 3.463 | 2.658 | 0.806 | 1.828 | 7.947 | 43.804 | 7.311 |
| 24 | FLJ13611 | null | 80006 | 80006 | hCG16824.4 | Hs.482301 | -3.457 | -3.064 | 0.393 | 1.828 | 7.616 | -1.756 | 7.616 |
| 25 | SFTPA2 | surfactant, pulmonary-associated protein A2 | 6436 | 6436 | hCG2025158 | Hs.464987 | 3.417 | 2.643 | 0.774 | 1.828 | 7.311 | 26.604 | 7.311 |
| 26 | WDR45 | WD repeat domain 45 | 11152 | 11152 | hCG1986580 | Hs.156701 | -3.396 | -3.004 | 0.393 | 2.742 | 10.545 | -1.763 | 10.545 |
| 27 | null | null | hCG1787594.2 | null | hCG1787594.2 | Hs.235795\|Hs.570761 | 3.393 | 2.627 | 0.766 | 2.742 | 10.154 | 2.048 | 8.844 |
| 28 | NGFRAP1L1 | NGFRAP1-like 1 | 340542 | 340542 | hCG1642311.3 | Hs.47209 | 3.38 | 2.614 | 0.766 | 2.742 | 9.792 | 4.566 | 8.844 |
| 29 | CTSE | cathepsin E | 1510 | 1510 | hCG18377.2 | Hs.1355 | 3.38 | 2.602 | 0.778 | 2.742 | 9.454 | 7.318 | 8.844 |
| 30 | SFTPB | surfactant, pulmonary-associated protein B | 6439 | 6439 | hCG34600.3 | Hs.512690 | 3.377 | 2.588 | 0.79 | 2.742 | 9.139 | 11.346 | 8.844 |
| 31 | PCMTD1 | protein-L-isoaspartate (D-aspartate) O-methyltransferase domain containing 1 | 115294 | 115294 | hCG27079.3 | Hs.308480 | 3.351 | 2.577 | 0.775 | 2.742 | 8.844 | 1.684 | 8.844 |
| 32 | EHMT2 | euchromatic histone-lysine N-methyltransferase 2 | 10919 | 10919 | hCG1811586.1 | Hs.520038 | -3.319 | -2.96 | 0.359 | 3.656 | 11.424 | -2.715 | 11.424 |
| 33 | IL1B | interleukin 1, beta | 3553 | 3553 | hCG16263.2 | Hs.126256 | 3.264 | 2.564 | 0.7 | 3.656 | 11.077 | 3.736 | 11.077 |
| 34 | FOS | v-fos FBJ murine osteosarcoma viral oncogene homolog | 2353 | 2353 | hCG22355.3 | Hs.568953\|Hs.25647 | 3.254 | 2.553 | 0.7 | 4.569 | 13.44 | 4.863 | 11.424 |
| 35 | CXCL3 | chemokine (C-X-C motif) ligand 3 | 2921 | 2921 | hCG16366.3 | Hs.89690 | 3.25 | 2.542 | 0.708 | 4.569 | 13.056 | 6.875 | 11.424 |
| 36 | MLLT4 | myeloid/lymphoid or mixed-lineage leukemia (trithorax homolog, Drosophila); translocated to, 4 | 4301 | 4301 | hCG2041523 | Hs.448059 | 3.214 | 2.532 | 0.682 | 4.569 | 12.693 | 1.965 | 11.424 |
| 37 | MGC17299 | null | 128218 | 128218 | hCG1644000.4 | Hs.104476 | 3.211 | 2.522 | 0.689 | 4.569 | 12.35 | 3.67 | 11.424 |
| 38 | FLJ21125 | null | 79680 | 79680 | hCG2002279.1 | Hs.105642 | 3.199 | 2.511 | 0.687 | 4.569 | 12.025 | 1.612 | 11.424 |
| 39 | MGC16943 | null | 112479 | 112479 | hCG38246.3 | Hs.248437 | -3.195 | -2.918 | 0.276 | 4.569 | 11.717 | -2.408 | 11.717 |
| 40 | SPINT2 | serine peptidase inhibitor, Kunitz type, 2 | 10653 | 10653 | hCG42856.3 | Hs.31439 | 3.194 | 2.502 | 0.692 | 4.569 | 11.424 | 3.365 | 11.424 |
| 41 | RDHE2 | null | 195814 | 195814 | hCG1787435.2 | Hs.170673 | 3.188 | 2.493 | 0.695 | 5.483 | 13.374 | 6.131 | 12.462 |
| 42 | LOC401431 | null | 401431 | 401431 | null | Hs.556097 | -3.179 | -2.885 | 0.295 | 5.483 | 13.056 | -2.553 | 12.752 |
| 43 | null | null | hCG1801741.2 | null | hCG1801741.2 | null | -3.156 | -2.852 | 0.304 | 5.483 | 12.752 | -2.66 | 12.752 |
| 44 | AQP4 | aquaporin 4 | 361 | 361 | hCG37174.3 | Hs.315369 | 3.147 | 2.484 | 0.663 | 5.483 | 12.462 | 8.626 | 12.462 |
| 45 | BOC | null | 91653 | 91653 | hCG17534.2 | Hs.556004 | -3.129 | -2.822 | 0.307 | 6.397 | 14.216 | -1.776 | 13.056 |
| 46 | CYP2B6 | cytochrome P450, family 2, subfamily B, polypeptide 6 | 1555 | 1555 | hCG1995522 | Hs.1360 | 3.124 | 2.475 | 0.65 | 6.397 | 13.907 | 4.09 | 12.544 |
| 47 | KIAA0703 | null | 9914 | 9914 | hCG39788.3 | Hs.6168 | 3.119 | 2.466 | 0.653 | 6.397 | 13.611 | 3.794 | 12.544 |
| 48 | DSP | desmoplakin | 1832 | 1832 | hCG14677.2 | Hs.519873 | 3.115 | 2.458 | 0.657 | 6.397 | 13.328 | 4.321 | 12.544 |
| 49 | CYP4B1 | cytochrome P450, family 4, subfamily B, polypeptide 1 | 1580 | 1580 | hCG22100.5 | Hs.436317 | 3.109 | 2.45 | 0.658 | 6.397 | 13.056 | 11.308 | 12.544 |
| 50 | BAIAP2L1 | BAI1-associated protein 2-like 1 | 55971 | 55971 | hCG41326.4 | Hs.567541 | 3.107 | 2.443 | 0.663 | 6.397 | 12.794 | 5.009 | 12.544 |
| 51 | CXCL5 | chemokine (C-X-C motif) ligand 5 | 6374 | 6374 | hCG16364.3 | Hs.89714 | 3.106 | 2.436 | 0.67 | 6.397 | 12.544 | 3.034 | 12.544 |
| 52 | KIAA1799 | null | 84455 | 84455 | hCG23452.4 | Hs.269768 | -3.1 | -2.796 | 0.304 | 7.311 | 14.06 | -1.769 | 13.056 |
| 53 | SGK | serum/glucocorticoid regulated kinase | 6446 | 6446 | hCG32737.3 | Hs.510078 | 3.083 | 2.428 | 0.655 | 7.311 | 13.795 | 2.573 | 13.293 |
| 54 | null | null | hCG1643044.2 | null | hCG1643044.2 | Hs.128395\|Hs.356460 | -3.076 | -2.773 | 0.303 | 7.311 | 13.539 | -2.403 | 13.056 |
| 55 | IL6 | interleukin 6 (interferon, beta 2) | 3569 | 3569 | hCG38231.4 | Hs.512234 | 3.066 | 2.42 | 0.646 | 7.311 | 13.293 | 6.385 | 13.293 |
| 56 | VARSL | valyl-tRNA synthetase like | 57176 | 57176 | hCG1788592.2 | Hs.102910 | -3.065 | -2.751 | 0.314 | 7.311 | 13.056 | -2.06 | 13.056 |
| 57 | TRIM23 | tripartite motif-containing 23 | 373 | 373 | hCG17860.4 | Hs.567237 | -3.062 | -2.73 | 0.332 | 8.225 | 14.43 | -1.608 | 14.06 |
| 58 | null | null | hCG2037001 | null | hCG2037001 | null | 3.056 | 2.413 | 0.643 | 8.225 | 14.181 | 3.682 | 13.484 |
| 59 | OSM | oncostatin M | 5008 | 5008 | hCG41316.3 | Hs.248156 | 3.042 | 2.406 | 0.637 | 8.225 | 13.941 | 2.487 | 13.484 |
| 60 | null | null | hCG2041353 | null | hCG2041353 | null | 3.037 | 2.399 | 0.638 | 8.225 | 13.708 | 1.964 | 13.484 |
| 61 | SNF1LK | SNF1-like kinase | 150094 | 150094 | hCG401294.2 | Hs.282113 | 3.037 | 2.392 | 0.644 | 8.225 | 13.484 | 3.292 | 13.484 |
| 62 | OR6K3 | olfactory receptor, family 6, subfamily K, member 3 | 391114 | 391114 | hCG1645091.2 | null | 3.022 | 2.386 | 0.636 | 9.139 | 14.74 | 2.384 | 14.28 |
| 63 | ATF3 | activating transcription factor 3 | 467 | 467 | hCG37734.3 | Hs.460 | 3.018 | 2.38 | 0.639 | 9.139 | 14.506 | 3.171 | 14.28 |

| | | | | | | | | | | | | | |
|---|---|---|---|---|---|---|---|---|---|---|---|---|---|
| 64 | RGS2 | regulator of G-protein signalling 2, 24kDa | 5997 | 5997 | hCG41052.3 | Hs.78944 | 3.017 | 2.372 | 0.645 | 9.139 | 14.28 | 3.436 | 14.28 |
| 65 | NKD1 | naked cuticle homolog 1 (Drosophila) | 85407 | 85407 | hCG24311.2 | Hs.298434 | -3.01 | -2.711 | 0.3 | 9.139 | 14.06 | -2.097 | 14.06 |
| 66 | DENND3 | DENN/MADD domain containing 3 | 22898 | 22898 | hCG20740.4 | Hs.18166 | 3.006 | 2.366 | 0.641 | 10.053 | 15.232 | 2.297 | 14.569 |
| 67 | ABCC3 | ATP-binding cassette, sub-family C (CFTR/MRP), member 3 | 8714 | 8714 | hCG29634.3 | Hs.463421 | 3.006 | 2.36 | 0.646 | 10.053 | 15.004 | 3.337 | 14.569 |
| 68 | CLDN4 | claudin 4 | 1364 | 1364 | null | Hs.520942 | 2.998 | 2.354 | 0.644 | 10.053 | 14.784 | 2.277 | 14.569 |
| 69 | null | null | hCG1820954.2 | null | hCG1820954.2 | null | 2.983 | 2.348 | 0.634 | 10.053 | 14.569 | 2.771 | 14.569 |
| 70 | TMC5 | transmembrane channel-like 5 | 79838 | 79838 | hCG38022.3 | Hs.115838 | 2.976 | 2.342 | 0.634 | 10.967 | 15.667 | 4.127 | 15.446 |
| 71 | FGFR4 | fibroblast growth factor receptor 4 | 2264 | 2264 | hCG1821375.1 | Hs.165950 | 2.963 | 2.337 | 0.626 | 10.967 | 15.446 | 1.915 | 15.446 |
| 72 | ST7L | suppression of tumorigenicity 7 like | 54879 | 54879 | hCG38462.3 | Hs.201921 | -2.962 | -2.692 | 0.269 | 11.881 | 16.501 | -2.032 | 15.429 |
| 73 | C9orf58 | chromosome 9 open reading frame 58 | 83543 | 83543 | hCG30196.2 | Hs.4944 | -2.962 | -2.675 | 0.286 | 11.881 | 16.275 | -4.931 | 15.429 |
| 74 | UROD | uroporphyrinogen decarboxylase | 7389 | 7389 | hCG17844.2 | Hs.78601 | -2.958 | -2.658 | 0.3 | 11.881 | 16.055 | -1.712 | 15.429 |
| 75 | ARMC5 | armadillo repeat containing 5 | 79798 | 79798 | hCG1772019.3 | Hs.121915 | -2.954 | -2.642 | 0.312 | 11.881 | 15.841 | -1.694 | 15.429 |
| 76 | null | null | ABI408610 | null | null | null | 2.95 | 2.331 | 0.618 | 11.881 | 15.632 | 1.918 | 15.632 |
| 77 | ZBTB17 | zinc finger and BTB domain containing 17 | 7709 | 7709 | hCG25370.3 | Hs.433764 | -2.945 | -2.628 | 0.317 | 11.881 | 15.429 | -1.64 | 15.429 |
| 78 | FLJ21839 | null | 60509 | 60509 | hCG1990058 | Hs.138207 | -2.93 | -2.614 | 0.316 | 12.794 | 16.403 | -1.772 | 16.403 |
| 79 | null | null | hCG1640549.4 | null | hCG1640549.4 | null | 2.929 | 2.326 | 0.603 | 12.794 | 16.196 | 4.124 | 15.993 |
| 80 | S100A9 | S100 calcium binding protein A9 (calgranulin B) | 6280 | 6280 | hCG15465.3 | Hs.112405 | 2.928 | 2.32 | 0.608 | 12.794 | 15.993 | 4.007 | 15.993 |
| 81 | null | null | hCG1818462.1 | null | hCG1818462.1 | null | 2.899 | 2.315 | 0.584 | 14.622 | 18.052 | 2.596 | 16.696 |
| 82 | SFTPG | surfactant associated protein G | 389376 | 389376 | hCG2006673 | Hs.211267 | 2.896 | 2.309 | 0.586 | 14.622 | 17.832 | 5.46 | 16.696 |
| 83 | null | null | hCG1790588.2 | null | hCG1790588.2 | null | -2.896 | -2.601 | 0.294 | 14.622 | 17.617 | -2.321 | 16.858 |
| 84 | LOC92270 | null | 92270 | 92270 | hCG1641198.2 | Hs.364045 | -2.895 | -2.588 | 0.307 | 14.622 | 17.407 | -2.262 | 16.858 |
| 85 | RGC32 | null | 28984 | 28984 | hCG32189.3 | Hs.507866 | 2.893 | 2.305 | 0.589 | 14.622 | 17.203 | 2.875 | 16.696 |
| 86 | null | null | hCG2000660 | null | hCG2000660 | Hs.569727 | -2.886 | -2.575 | 0.31 | 14.622 | 17.003 | -2.113 | 16.858 |
| 87 | EIF1AY | eukaryotic translation initiation factor 1A, Y-linked | 9086 | 9086 | hCG37922.4 | Hs.584171\|Hs.461178 | 2.884 | 2.299 | 0.585 | 14.622 | 16.807 | 10.888 | 16.696 |
| 88 | KPTN | kaptin (actin binding protein) | 11133 | 11133 | hCG20550.3 | Hs.25441 | -2.88 | -2.563 | 0.317 | 15.536 | 17.655 | -1.757 | 16.858 |
| 89 | TOB1 | transducer of ERBB2, 1 | 10140 | 10140 | hCG1639758.3 | Hs.531550 | 2.878 | 2.295 | 0.584 | 15.536 | 17.456 | 1.85 | 16.696 |
| 90 | SKIV2L2 | superkiller viralicidic activity 2-like 2 (S. cerevisiae) | 23517 | 23517 | hCG40991.3 | Hs.274531 | -2.877 | -2.552 | 0.325 | 15.536 | 17.262 | -1.554 | 16.858 |
| 91 | null | null | hCG2042709 | null | hCG2042709 | Hs.530461 | -2.874 | -2.541 | 0.333 | 15.536 | 17.073 | -2.217 | 16.858 |
| 92 | DKFZp781N1041 | null | 387712 | 387712 | null | Hs.372457 | 2.863 | 2.29 | 0.573 | 15.536 | 16.887 | 2.507 | 16.696 |
| 93 | null | null | hCG1993095.1 | null | hCG1993095.1 | null | 2.858 | 2.285 | 0.573 | 16.45 | 17.688 | 3.554 | 16.696 |
| 94 | null | null | hCG2042092 | null | hCG2042092 | null | 2.853 | 2.281 | 0.573 | 16.45 | 17.5 | 1.544 | 16.696 |
| 95 | TUFM | Tu translation elongation factor, mitochondrial | 7284 | 7284 | hCG1747191.2 | Hs.12084 | -2.85 | -2.529 | 0.321 | 16.45 | 17.316 | -1.775 | 16.858 |
| 96 | MAL2 | mal, T-cell differentiation protein 2 | 114569 | 114569 | hCG1653390.2 | Hs.201083 | 2.848 | 2.276 | 0.572 | 17.364 | 18.087 | 5.943 | 16.696 |
| 97 | BAI2 | brain-specific angiogenesis inhibitor 2 | 576 | 576 | hCG41602.3 | Hs.524138 | -2.847 | -2.518 | 0.328 | 17.364 | 17.901 | -3.149 | 16.858 |
| 98 | LCP2 | lymphocyte cytosolic protein 2 (SH2 domain containing leukocyte protein of 76kDa) | 3937 | 3937 | hCG37753.3 | Hs.304475 | 2.846 | 2.271 | 0.575 | 17.364 | 17.718 | 2.654 | 16.696 |
| 99 | ROS1 | v-ros UR2 sarcoma virus oncogene homolog 1 (avian) | 6098 | 6098 | hCG1811669.2 | Hs.1041 | 2.845 | 2.266 | 0.579 | 17.364 | 17.539 | 4.322 | 16.696 |
| 100 | THBS4 | thrombospondin 4 | 7060 | 7060 | hCG2015975 | Hs.211426 | -2.843 | -2.508 | 0.335 | 17.364 | 17.364 | -7.001 | 16.858 |
| 101 | UNQ5810 | null | 388218 | 388218 | hCG1657150.4 | Hs.115838 | 2.839 | 2.261 | 0.577 | 17.364 | 17.192 | 4.269 | 16.696 |
| 102 | FLJ39660 | null | 284992 | 284992 | hCG2043053.1 | Hs.132519 | -2.834 | -2.499 | 0.335 | 17.364 | 17.023 | -2.39 | 16.858 |
| 103 | KIAA0372 | KIAA0372 | 9652 | 9652 | hCG1811113.2 | Hs.482868 | -2.828 | -2.489 | 0.338 | 17.364 | 16.858 | -1.509 | 16.858 |
| 104 | GPRC5A | G protein-coupled receptor, family C, group 5, member A | 9052 | 9052 | hCG27693.3 | Hs.194691 | 2.827 | 2.257 | 0.57 | 17.364 | 16.696 | 4.316 | 16.696 |
| 105 | C14orf4 | chromosome 14 open reading frame 4 | 64207 | 64207 | hCG21245.3 | Hs.179260 | 2.825 | 2.252 | 0.572 | 18.278 | 17.407 | 1.892 | 17.184 |
| 106 | null | null | hCG2040471 | null | hCG2040471 | null | 2.823 | 2.248 | 0.574 | 18.278 | 17.243 | 2.131 | 17.184 |
| 107 | DKFZp686E2433 | null | 345462 | 345462 | hCG2041603 | Hs.445740 | -2.811 | -2.48 | 0.331 | 19.192 | 17.936 | -1.718 | 17.135 |
| 108 | CEBPE | CCAAT/enhancer binding protein (C/EBP), epsilon | 1053 | 1053 | hCG41750.2 | Hs.558308 | 2.809 | 2.244 | 0.566 | 19.192 | 17.77 | 1.553 | 17.184 |
| 109 | CAV2 | caveolin 2 | 858 | 858 | hCG39087.4 | Hs.212332 | 2.808 | 2.239 | 0.569 | 19.192 | 17.607 | 2.324 | 17.184 |
| 110 | PON2 | paraoxonase 2 | 5445 | 5445 | hCG1640820.2 | Hs.530077 | 2.806 | 2.235 | 0.57 | 19.192 | 17.447 | 1.693 | 17.184 |
| 111 | DDX3X | DEAD (Asp-Glu-Ala-Asp) box polypeptide 3, X-linked | 1654 | 1654 | hCG19318.3 | Hs.380774 | 2.801 | 2.231 | 0.57 | 19.192 | 17.29 | 2.131 | 17.184 |
| 112 | CDKL3 | cyclin-dependent kinase-like 3 | 51265 | 51265 | hCG1980962.1 | Hs.105818 | -2.8 | -2.471 | 0.329 | 19.192 | 17.135 | -1.922 | 17.135 |
| 113 | ZNF300 | zinc finger protein 300 | 91975 | 91975 | hCG1980728 | Hs.134885 | -2.796 | -2.462 | 0.333 | 20.106 | 17.793 | -2.462 | 17.793 |
| 114 | DUSP1 | dual specificity phosphatase 1 | 1843 | 1843 | hCG41245.2 | Hs.171695 | 2.793 | 2.227 | 0.566 | 20.106 | 17.637 | 2.83 | 17.184 |
| 115 | ABCA3 | ATP-binding cassette, sub-family A (ABC1), member 3 | 21 | 21 | hCG41022.5 | Hs.26630 | 2.791 | 2.223 | 0.568 | 20.106 | 17.483 | 5.183 | 17.184 |
| 116 | PEBP4 | null | 157310 | 157310 | hCG15999.3 | Hs.491242 | 2.79 | 2.219 | 0.571 | 20.106 | 17.332 | 4.073 | 17.184 |
| 117 | null | null | hCG1998836.1 | null | hCG1998836.1 | null | 2.788 | 2.215 | 0.573 | 20.106 | 17.184 | 1.506 | 17.184 |
| 118 | TYRP1 | tyrosinase-related protein 1 | 7306 | 7306 | hCG27256.4 | Hs.270279 | 2.781 | 2.211 | 0.571 | 21.02 | 17.813 | 3.868 | 17.663 |
| 119 | LPP | LIM domain containing preferred translocation partner in lipoma | 4026 | 4026 | hCG17155.3 | Hs.444362 | 2.772 | 2.207 | 0.565 | 21.02 | 17.663 | 1.718 | 17.663 |
| 120 | ANKRD32 | ankyrin repeat domain 32 | 84250 | 84250 | hCG2016110 | Hs.285055\|Hs.482853 | -2.769 | -2.454 | 0.315 | 21.933 | 18.278 | -1.849 | 17.832 |
| 121 | null | null | hCG1817888.2 | null | hCG1817888.2 | Hs.498939 | 2.766 | 2.203 | 0.564 | 21.933 | 18.127 | 1.394 | 17.688 |
| 122 | IDH3B | isocitrate dehydrogenase 3 (NAD+) beta | 3420 | 3420 | hCG39616.3 | Hs.436405 | -2.765 | -2.446 | 0.32 | 21.933 | 17.978 | -1.733 | 17.832 |
| 123 | KIAA0649 | KIAA0649 | 9858 | 9858 | hCG27241.3 | Hs.533260 | -2.759 | -2.438 | 0.321 | 21.933 | 17.832 | -1.901 | 17.832 |
| 124 | TREM1 | triggering receptor expressed on myeloid cells 1 | 54210 | 54210 | hCG32869.2 | Hs.283022 | 2.758 | 2.199 | 0.559 | 21.933 | 17.688 | 6.164 | 17.688 |
| 125 | NR4A3 | nuclear receptor subfamily 4, group A, member 3 | 8013 | 8013 | hCG28754.3 | Hs.279522 | 2.749 | 2.196 | 0.553 | 22.847 | 18.278 | 4.115 | 17.711 |
| 126 | ARF6 | ADP-ribosylation factor 6 | 382 | 382 | hCG2013574 | Hs.525330 | 2.746 | 2.192 | 0.554 | 22.847 | 18.133 | 1.728 | 17.711 |
| 127 | KIAA1772 | KIAA1772 | 80000 | 80000 | hCG1811231.5 | Hs.54838 | -2.745 | -2.43 | 0.315 | 22.847 | 17.99 | -1.862 | 17.849 |
| 128 | ZNF228 | zinc finger protein 228 | 7771 | 7771 | hCG20223.4 | Hs.48589 | -2.744 | -2.423 | 0.322 | 22.847 | 17.849 | -1.639 | 17.849 |
| 129 | CLDN18 | claudin 18 | 51208 | 51208 | hCG2022628 | Hs.240182 | 2.743 | 2.189 | 0.555 | 22.847 | 17.711 | 6.311 | 17.711 |

| | | | | | | | | | | | | | |
|---|---|---|---|---|---|---|---|---|---|---|---|---|---|
| 130 IL17D | interleukin 17D | 53342 | 53342 | hCG31110.3 | Hs.130652 | -2.741 | -2.415 | 0.326 | 23.761 | 18.278 | -3.281 | 18.001 |
| 131 null | null | hCG2040182 | null | hCG2040182 | null | 2.738 | 2.185 | 0.553 | 23.761 | 18.138 | 1.595 | 18.138 |
| 132 FAM40A | family with sequence similarity 40, member A | 85369 | 85369 | hCG40257.3 | Hs.169577 | -2.738 | -2.409 | 0.329 | 23.761 | 18.001 | -1.467 | 18.001 |
| 133 LOC92659 | null | 92659 | 92659 | hCG1987587 | Hs.336958 | -2.73 | -2.401 | 0.328 | 24.675 | 18.553 | -2.335 | 18.543 |
| 134 FOSB | FBJ murine osteosarcoma viral oncogene homolog B | 2354 | 2354 | hCG20725.3 | Hs.75678 | 2.726 | 2.182 | 0.544 | 24.675 | 18.414 | 2.514 | 18.414 |
| 135 null | null | hCG2041761 | null | hCG2041761 | null | 2.716 | 2.178 | 0.538 | 25.589 | 18.955 | 2.015 | 18.815 |
| 136 RPS4Y1 | ribosomal protein S4, Y-linked 1 | 6192 | 6192 | hCG1988058 | Hs.282376 | 2.715 | 2.174 | 0.54 | 25.589 | 18.815 | 46.164 | 18.815 |
| 137 null | null | hCG1640591.2 | null | hCG1640591.2 | null | -2.714 | -2.394 | 0.32 | 25.589 | 18.678 | -1.763 | 18.543 |
| 138 TGIF2 | TGFB-induced factor 2 (TALE family homeobox) | 60436 | 60436 | hCG31789.3 | Hs.292281 | -2.714 | -2.387 | 0.327 | 25.589 | 18.543 | -1.725 | 18.543 |
| 139 BRD8 | bromodomain containing 8 | 10902 | 10902 | hCG39941.2 | Hs.519337 | -2.703 | -2.38 | 0.323 | 26.503 | 19.067 | -1.711 | 18.887 |
| 140 RBM35B | RNA binding motif protein 35B | 80004 | 80004 | hCG28119.2 | Hs.436585 | 2.702 | 2.171 | 0.532 | 27.417 | 19.583 | 4.748 | 18.99 |
| 141 MMP7 | matrix metallopeptidase 7 (matrilysin, uterine) | 4316 | 4316 | hCG1640914.2 | Hs.2256 | 2.699 | 2.167 | 0.532 | 27.417 | 19.445 | 5.138 | 18.99 |
| 142 TACSTD2 | tumor-associated calcium signal transducer 2 | 4070 | 4070 | hCG22165.2 | Hs.23582 | 2.696 | 2.164 | 0.532 | 27.417 | 19.308 | 4.867 | 18.99 |
| 143 TNFRSF10D | tumor necrosis factor receptor superfamily, member 10d, decoy with truncated death domain | 8793 | 8793 | hCG1645071.3 | Hs.213467 | 2.692 | 2.16 | 0.532 | 27.417 | 19.173 | 2.835 | 18.99 |
| 144 ADCK4 | aarF domain containing kinase 4 | 79934 | 79934 | hCG1995823.1 | Hs.130712 | -2.691 | -2.374 | 0.317 | 27.417 | 19.039 | -1.509 | 18.887 |
| 145 SFTPA1 | surfactant, pulmonary-associated protein A1 | 6435 | 6435 | hCG2025128 | Hs.465126 | 2.69 | 2.157 | 0.533 | 28.331 | 19.538 | 12.129 | 18.99 |
| 146 LOC284591 | null | 284591 | 284591 | hCG2038553\|hCG1995881 | Hs.133183 | -2.688 | -2.368 | 0.321 | 28.331 | 19.405 | -1.836 | 18.887 |
| 147 MGC33657 | null | 200373 | 200373 | hCG1804625.3 | null | 2.688 | 2.154 | 0.534 | 28.331 | 19.273 | 1.537 | 18.99 |
| 148 SLC11A1 | solute carrier family 11 (proton-coupled divalent metal ion transporters), member 1 | 6556 | 6556 | hCG16301.2 | Hs.471393 | 2.685 | 2.151 | 0.534 | 28.331 | 19.142 | 2.717 | 18.99 |
| 149 SSTR2 | somatostatin receptor 2 | 6752 | 6752 | hCG1641451.1 | Hs.514451 | -2.684 | -2.361 | 0.323 | 28.331 | 19.014 | -2.298 | 18.887 |
| 150 null | null | hCG1996962 | null | hCG1996962 | Hs.368518 | -2.684 | -2.355 | 0.328 | 28.331 | 18.887 | -1.941 | 18.887 |
| 151 MYH9 | myosin, heavy polypeptide 9, non-muscle | 4627 | 4627 | hCG41454.3 | Hs.474751 | 2.682 | 2.147 | 0.535 | 29.245 | 19.367 | 2.033 | 18.99 |
| 152 BHLHB2 | basic helix-loop-helix domain containing, class B, 2 | 8553 | 8553 | hCG20358.2 | Hs.171825 | 2.682 | 2.145 | 0.538 | 29.245 | 19.24 | 2.132 | 18.99 |
| 153 GATA6 | GATA binding protein 6 | 2627 | 2627 | hCG37191.2 | Hs.514746 | 2.682 | 2.141 | 0.541 | 29.245 | 19.114 | 4.338 | 18.99 |
| 154 null | null | hCG1820902.1 | null | hCG1820902.1 | null | 2.673 | 2.138 | 0.535 | 29.245 | 18.99 | 1.913 | 18.99 |
| 155 FLJ22688 | null | 80199 | 80199 | hCG21121.3 | Hs.288800 | -2.666 | -2.35 | 0.316 | 30.158 | 19.457 | -1.903 | 19.457 |
| 156 C1orf116 | chromosome 1 open reading frame 116 | 79098 | 79098 | hCG1723946.2 | Hs.32417 | 2.665 | 2.135 | 0.53 | 30.158 | 19.332 | 5.578 | 19.209 |
| 157 GLS | glutaminase | 2744 | 2744 | hCG25795.2 | Hs.116448 | 2.664 | 2.132 | 0.532 | 30.158 | 19.209 | 1.739 | 19.209 |
| 158 ZFYVE1 | zinc finger, FYVE domain containing 1 | 53349 | 53349 | hCG21522.3 | Hs.335106 | -2.656 | -2.343 | 0.313 | 31.072 | 19.666 | -1.398 | 19.666 |
| 159 NAPSA | napsin A aspartic peptidase | 9476 | 9476 | hCG22926.2 | Hs.512843 | 2.655 | 2.129 | 0.525 | 31.072 | 19.542 | 3.232 | 19.542 |
| 160 TTTY15 | testis-specific transcript, Y-linked 15 | 64595 | 64595 | hCG2040553\|hCG2039064 | Hs.433656 | 2.651 | 2.126 | 0.525 | 31.986 | 19.991 | 6.304 | 19.745 |
| 161 DDX20 | DEAD (Asp-Glu-Ala-Asp) box polypeptide 20 | 11218 | 11218 | hCG38167.3 | Hs.485810 | -2.649 | -2.337 | 0.311 | 31.986 | 19.867 | -1.735 | 19.867 |
| 162 NFKBIZ | nuclear factor of kappa light polypeptide gene enhancer in B-cells inhibitor, zeta | 64332 | 64332 | hCG38701.5 | Hs.319171 | 2.648 | 2.124 | 0.524 | 31.986 | 19.745 | 2.464 | 19.745 |
| 163 AP2B1 | adaptor-related protein complex 2, beta 1 subunit | 163 | 163 | hCG1992160 | Hs.514819 | -2.644 | -2.331 | 0.313 | 32.9 | 20.184 | -1.489 | 19.939 |
| 164 VPS16 | vacuolar protein sorting 16 (yeast) | 64601 | 64601 | hCG2039972 | Hs.269577 | -2.643 | -2.325 | 0.318 | 32.9 | 20.061 | -1.523 | 19.939 |
| 165 CENPH | centromere protein H | 64946 | 64946 | hCG27171.3 | Hs.200395 | -2.639 | -2.32 | 0.32 | 32.9 | 19.939 | -2.103 | 19.939 |
| 166 KIFC3 | kinesin family member C3 | 3801 | 3801 | hCG1795607.2 | Hs.23131 | 2.638 | 2.121 | 0.517 | 32.9 | 19.819 | 2.75 | 19.819 |
| 167 MAP3K5 | mitogen-activated protein kinase kinase kinase 5 | 4217 | 4217 | hCG33356.3 | Hs.186486 | 2.637 | 2.118 | 0.519 | 33.814 | 20.248 | 2.292 | 20.248 |
| 168 null | null | hCG1650256.3 | null | hCG1650256.3 | null | -2.632 | -2.314 | 0.318 | 33.814 | 20.127 | -1.763 | 20.074 |
| 169 PPM1G | protein phosphatase 1G (formerly 2C), magnesium-dependent, gamma isoform | 5496 | 5496 | hCG21229.3 | Hs.17883 | -2.631 | -2.309 | 0.322 | 34.728 | 20.549 | -1.471 | 20.074 |
| 170 AMH | anti-Mullerian hormone | 268 | 268 | hCG2039529 | Hs.112432 | -2.63 | -2.304 | 0.326 | 34.728 | 20.428 | -2.64 | 20.074 |
| 171 PPT2 | palmitoyl-protein thioesterase 2 | 9374 | 9374 | hCG1999928.1 | Hs.332138 | -2.629 | -2.298 | 0.331 | 34.728 | 20.309 | -1.557 | 20.074 |
| 172 SYT11 | synaptotagmin XI | 23208 | 23208 | hCG17193.3 | Hs.32984 | -2.629 | -2.293 | 0.336 | 34.728 | 20.191 | -2.212 | 20.074 |
| 173 FKSG14 | null | 64105 | 64105 | hCG1747136.3 | Hs.529778 | -2.629 | -2.289 | 0.34 | 34.728 | 20.074 | -2.35 | 20.074 |
| 174 CD69 | CD69 antigen (p60, early T-cell activation antigen) | 969 | 969 | hCG38142.3 | Hs.208854 | 2.618 | 2.115 | 0.503 | 36.556 | 21.009 | 3.137 | 20.889 |
| 175 CYorf15B | chromosome Y open reading frame 15B | 84663 | 84663 | hCG1987333 | Hs.145010 | 2.616 | 2.112 | 0.504 | 36.556 | 20.889 | 6.819 | 20.889 |
| 176 LNPEP | leucyl/cystinyl aminopeptidase | 4012 | 4012 | hCG1735557.1 | Hs.554761 | -2.606 | -2.284 | 0.323 | 37.47 | 21.29 | -1.561 | 21.206 |
| 177 ETV5 | ets variant gene 5 (ets-related molecule) | 2119 | 2119 | null | Hs.43697 | 2.601 | 2.11 | 0.492 | 38.383 | 21.686 | 1.919 | 21.474 |
| 178 null | null | hCG2024913 | null | hCG2024913 | null | 2.601 | 2.107 | 0.494 | 38.383 | 21.564 | 1.907 | 21.474 |
| 179 KIAA0664 | KIAA0664 | 23277 | 23277 | hCG32976.4 | Hs.22616 | -2.601 | -2.279 | 0.322 | 38.383 | 21.443 | -1.899 | 21.206 |
| 180 RAD54L | RAD54-like (S. cerevisiae) | 8438 | 8438 | hCG22090.2 | Hs.523220 | -2.6 | -2.274 | 0.326 | 38.383 | 21.324 | -2.275 | 21.206 |
| 181 MGC33648 | null | 133383 | 133383 | hCG40616.3 | Hs.85950 | -2.599 | -2.269 | 0.33 | 38.383 | 21.206 | -2.11 | 21.206 |
| 182 ITPR1 | inositol 1,4,5-triphosphate receptor, type 1 | 3708 | 3708 | hCG21731.2 | Hs.567295 | 2.59 | 2.104 | 0.486 | 39.297 | 21.592 | 2.164 | 21.474 |
| 183 CXCL1 | chemokine (C-X-C motif) ligand 1 (melanoma growth stimulating activity, alpha) | 2919 | 2919 | hCG16368.2 | Hs.789 | 2.59 | 2.101 | 0.489 | 39.297 | 21.474 | 2.206 | 21.474 |
| 184 CDH1 | cadherin 1, type 1, E-cadherin (epithelial) | 999 | 999 | hCG28201.2 | Hs.461086 | 2.584 | 2.099 | 0.485 | 40.211 | 21.854 | 6.424 | 21.854 |
| 185 STK11IP | serine/threonine kinase 11 interacting protein | 114790 | 114790 | hCG16070.3 | Hs.22410 | -2.58 | -2.264 | 0.316 | 40.211 | 21.736 | -1.48 | 21.736 |
| 186 LRRK2 | leucine-rich repeat kinase 2 | 120892 | 120892 | hCG1775001.3 | Hs.187636 | 2.579 | 2.096 | 0.483 | 41.125 | 22.11 | 2.817 | 22.11 |
| 187 null | null | hCG2016784 | null | hCG2016784 | Hs.453331 | 2.569 | 2.093 | 0.476 | 42.039 | 22.481 | 2.285 | 22.481 |
| 188 THOP1 | thimet oligopeptidase 1 | 7064 | 7064 | hCG25247.3 | Hs.78769 | -2.565 | -2.259 | 0.306 | 42.953 | 22.847 | -1.981 | 22.726 |
| 189 PDZRN4 | PDZ domain containing RING finger 4 | 29951 | 29951 | hCG1997488 | Hs.380044 | -2.565 | -2.255 | 0.31 | 42.953 | 22.726 | -4.065 | 22.726 |
| 190 VIPR1 | vasoactive intestinal peptide receptor 1 | 7433 | 7433 | hCG28852.4 | Hs.348500 | 2.564 | 2.091 | 0.474 | 42.953 | 22.607 | 3.992 | 22.607 |
| 191 ARHGAP8\|LOC553158 | Rho GTPase activating protein 8 | 23779 | 23779\|553158 | null | Hs.102336 | 2.559 | 2.088 | 0.471 | 43.867 | 22.967 | 3.66 | 22.612 |
| 192 HIST1H4A | histone 1, H4a | 8359 | 8359 | hCG1643898.2 | Hs.248178 | -2.557 | -2.25 | 0.307 | 43.867 | 22.847 | -2.151 | 22.847 |
| 193 BTG2 | BTG family, member 2 | 7832 | 7832 | hCG1640923.2 | Hs.519162 | 2.556 | 2.085 | 0.471 | 43.867 | 22.729 | 2.426 | 22.612 |
| 194 DIP | null | 23151 | 23151 | hCG41796.3 | Hs.475150 | 2.556 | 2.082 | 0.474 | 43.867 | 22.612 | 1.825 | 22.612 |
| 195 ANKRD13B | ankyrin repeat domain 13B | 124930 | 124930 | hCG29086.2 | Hs.334715 | -2.541 | -2.246 | 0.295 | 46.609 | 23.902 | -1.969 | 23.488 |

| | | | | | | | | | | | | | |
|---|---|---|---|---|---|---|---|---|---|---|---|---|---|
| 196 | null | null | hCG1645220.1 | null | hCG1645220.1 | null | -2.538 | -2.242 | 0.296 | 46.609 | 23.78 | -2.52 | 23.488 |
| 197 | ATRX | alpha thalassemia/mental retardation syndrome X-linked (RAD54 homolog, S. cerevisiae) | 546 | 546 | hCG20035.4 | Hs.533526\|Hs.571758 | 2.536 | 2.079 | 0.457 | 47.522 | 24.123 | 1.92 | 23.513 |
| 198 | MIDN | midnolin | 90007 | 90007 | hCG21588.3 | Hs.465529 | 2.536 | 2.077 | 0.459 | 47.522 | 24.001 | 1.825 | 23.513 |
| 199 | LOC400506 | null | 400506 | 400506 | hCG38020.2 | Hs.300404 | -2.535 | -2.238 | 0.297 | 47.522 | 23.881 | -1.642 | 23.488 |
| 200 | LOC148413\|LOC440746 | null | 440746 | 148413\|440746 | hCG20425.3 | Hs.529943\|Hs.515698 | -2.534 | -2.234 | 0.3 | 47.522 | 23.761 | -1.532 | 23.488 |
| 201 | STC1 | stanniocalcin 1 | 6781 | 6781 | hCG16685.3 | Hs.25590 | 2.532 | 2.074 | 0.458 | 48.436 | 24.098 | 2.116 | 23.513 |
| 202 | TIMM44 | translocase of inner mitochondrial membrane 44 homolog (yeast) | 10469 | 10469 | hCG22555.3 | Hs.465784 | -2.529 | -2.23 | 0.3 | 48.436 | 23.978 | -1.55 | 23.488 |
| 203 | null | null | hCG1994865 | null | hCG1994865 | Hs.533526 | 2.529 | 2.071 | 0.457 | 48.436 | 23.86 | 1.833 | 23.513 |
| 204 | null | null | hCG1778862.2 | null | hCG1778862.2 | null | -2.527 | -2.226 | 0.301 | 48.436 | 23.743 | -1.688 | 23.488 |
| 205 | LOC131873 | null | 131873 | 131873 | null | Hs.477571 | 2.527 | 2.069 | 0.458 | 48.436 | 23.627 | 4.484 | 23.513 |
| 206 | ZFP36 | zinc finger protein 36, C3H type, homolog (mouse) | 7538 | 7538 | hCG43352.3 | Hs.534052 | 2.526 | 2.066 | 0.459 | 48.436 | 23.513 | 2.709 | 23.513 |
| 207 | FOXD1 | forkhead box D1 | 2297 | 2297 | hCG37843.4 | Hs.519385 | -2.521 | -2.221 | 0.3 | 49.35 | 23.841 | -3.298 | 23.488 |
| 208 | null | null | hCG1774273.1 | null | hCG1774273.1 | null | -2.521 | -2.217 | 0.304 | 50.264 | 24.165 | -1.905 | 23.488 |
| 209 | FLJ36032 | null | 284485 | 284485 | hCG16929.3 | Hs.297967 | -2.52 | -2.213 | 0.307 | 50.264 | 24.05 | -1.892 | 23.488 |
| 210 | JUNB | jun B proto-oncogene | 3726 | 3726 | hCG172481.3 | Hs.25292 | 2.519 | 2.064 | 0.455 | 50.264 | 23.935 | 2.308 | 23.822 |
| 211 | PERP | PERP, TP53 apoptosis effector | 64065 | 64065 | hCG17829.4 | Hs.520421 | 2.519 | 2.061 | 0.458 | 50.264 | 23.822 | 2.395 | 23.822 |

**Supplementary Figure 1.** Unsupervised hierarchical clustering of all tumours based on the top-210 significant genes differentially expressed between metastases and primary tumours, identified by SAM analysis (Namløs et al.)

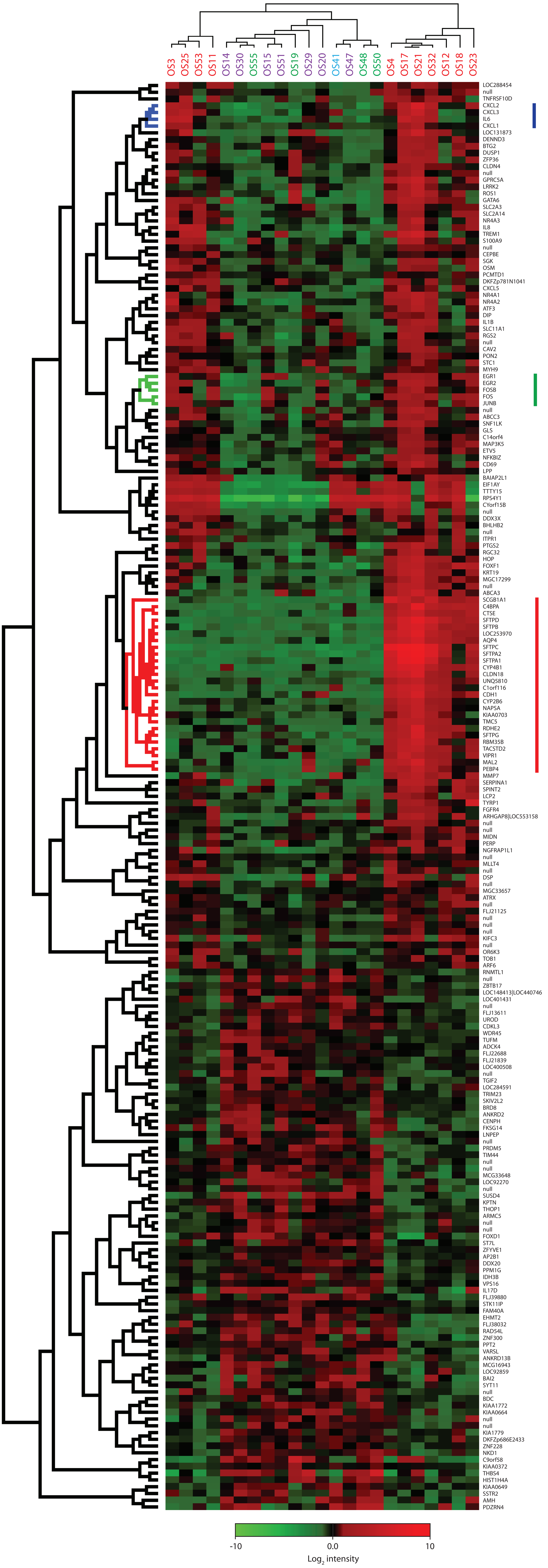

Supplement: Supplementary file 1 — Supplementary Table 1: Top-210 genes differentially expressed between primary tumours that did and did not develop into metastasis by SAM analysis. Supplementary Figure 1: Unsupervised hierarchical clustering of all tumours based on the top-210 significant genes differentially expressed between metastases and primary tumours, identified by SAM analysis. [file 639038.f1.pdf]
