# Supplementary material for: Global Gene Expression Profiling of Human Osteosarcomas Reveals Metastasis-Associated Chemokine Pattern
Source: Sarcoma. 2012 Feb 28;2012:639038. doi: 10.1155/2012/639038 (PMC3324929; doi:10.1155/2012/639038)
Supplement: Supplementary file 2 [file 639038.f2.pdf]

Supplementary Figure 1. Unsupervised hierarchical clustering of all tumours based on the top-210 significant genes differentially expressed between metastases and primary tumours, identified by SAM analysis (Namløs et al.)

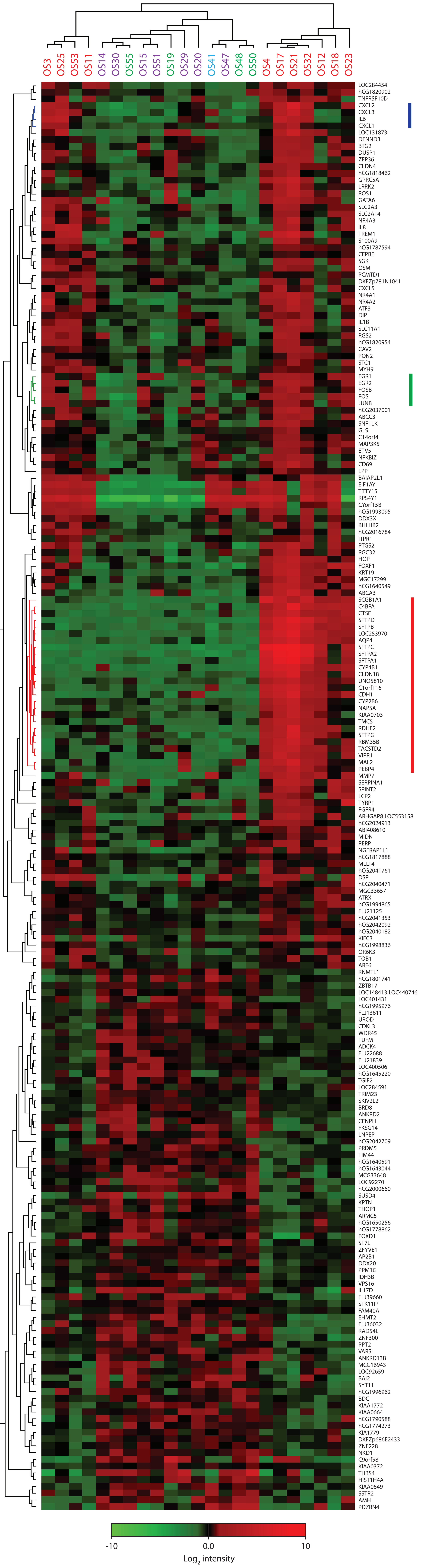

Supplementary Table 1. Top-210 genes differentially expressed between primary tumours that did and did not develop into metastasis by SAM analysis (Namløs et al.)

Number	Gene_Symbol	Gene_Name	Primary_Gene_ID	Entrez Gene ID	Celera Gene ID	UniGene ID	d[i]	de[i]	Delta[i]	FSN[i]	FDR[i]	Fold Change	q-val[i]
1	LOC284454	null	284454	284454	hCG2001068	Hs.436426	4.918	3.649	1.269	0	0	3.528	0
2	CXCL2	chemokine (C-X-C motif) ligand 2	2920	2920	hCG16361.2	Hs.75765	4.559	3.376	1.183	0	0	13.114	0
3	PTGS2	prostaglandin-endoperoxide synthase 2 (prostaglandin G/H synthase and cyclooxygenase)	5743	5743	hCG39885.3	Hs.196384	4.337	3.234	1.103	0	0	7.841	0
4	FOXF1	forkhead box F1	2294	2294	hCG17050.2	Hs.155591	4.155	3.142	1.014	0	0	9.416	0
5	RNMTL1	RNA methyltransferase like 1	55178	55178	hCG32500.2	Hs.182729	-4.119	-3.647	0.472	0	0	-1.969	0
6	SLC2A3	solute carrier family 2 (facilitated glucose transporter), member 3	6515	6515	hCG22218.2	Hs.419240	4.074	3.067	1.007	0	0	3.912	0
7	SLC2A14	solute carrier family 2 (facilitated glucose transporter), member 14	144195	144195	hCG1730826.1	Hs.210227	4.07	3.015	1.055	0	0	2.681	0
8	SCGB1A1	secretoglobin, family 1A, member 1 (uteroglobin)	7356	7356	hCG39843.2	Hs.523732	3.999	2.965	1.035	0	0	11.506	0
9	KRT19	keratin 19	3880	3880	hCG2043341	Hs.514167	3.976	2.921	1.056	0	0	5.473	0
10	IL8	interleukin 8	3576	3576	hCG16372.3	Hs.624	3.9	2.884	1.016	0	0	6.508	0
11	HOP	null	84525	84525	null	Hs.121443	3.857	2.852	1.005	0	0	6.389	0
12	SUSD4	sushi domain containing 4	55061	55061	hCG23752.3	Hs.497841	-3.778	-3.394	0.384	0.914	7.616	-4.155	6.093
13	NR4A1	nuclear receptor subfamily 4, group A, member 1	3164	3164	hCG37966.3	Hs.524430	3.769	2.824	0.945	0.914	7.03	4.888	4.81
14	EGR1	early growth response 1	1958	1958	hCG18777.1	Hs.326035	3.748	2.797	0.95	0.914	6.528	4.201	4.81
15	PRDM5	PR domain containing 5	11107	11107	hCG38692.3	Hs.132593	-3.739	-3.239	0.499	0.914	6.093	-2.266	6.093
16	SERPINA1	serpin peptidase inhibitor, clade A (alpha-1 antiproteinase, antitrypsin), member 1	5265	5265	hCG2029168.1	Hs.525557	3.705	2.772	0.933	0.914	5.712	6.586	4.81
17	C4BPA	complement component 4 binding protein, alpha	722	722	hCG1986997	Hs.1012	3.664	2.748	0.916	0.914	5.376	13.41	4.81
18	SFTPD	surfactant, pulmonary-associated protein D	6441	6441	hCG1993456	Hs.253495	3.601	2.729	0.872	0.914	5.077	17.991	4.81
19	EGR2	early growth response 2 (Krox-20 homolog, Drosophila)	1959	1959	hCG40234.2	Hs.1395	3.59	2.709	0.882	0.914	4.81	4.577	4.81
20	LOC253970	null	253970	253970	hCG2013488	Hs.509165	3.531	2.691	0.84	1.828	9.139	12.973	7.311
21	NR4A2	nuclear receptor subfamily 4, group A, member 2	4929	4929	hCG38357.2	Hs.165258	3.513	2.674	0.84	1.828	8.704	5.224	7.311
22	null	null	hCG1995976	null	hCG1995976	Hs.313856	-3.498	-3.137	0.36	1.828	8.308	-1.91	7.616
23	SFTPC	surfactant, pulmonary-associated protein C	6440	6440	hCG23794.3	Hs.1074	3.463	2.658	0.806	1.828	7.947	43.804	7.311
24	FLJ13611	null	80006	80006	hCG16824.4	Hs.482301	-3.457	-3.064	0.393	1.828	7.616	-1.756	7.616
25	SFTPA2	surfactant, pulmonary-associated protein A2	6436	6436	hCG2025158	Hs.464987	3.417	2.643	0.774	1.828	7.311	26.604	7.311
26	WDR45	WD repeat domain 45	11152	11152	hCG1986580	Hs.156701	-3.396	-3.004	0.393	2.742	10.545	-1.763	10.545
27	null	null	hCG1787594.2	null	hCG1787594.2	Hs.235795|Hs.570761	3.393	2.627	0.766	2.742	10.154	2.048	8.844
28	NGFRAP1L1	NGFRAP1-like 1	340542	340542	hCG1642311.3	Hs.47209	3.38	2.614	0.766	2.742	9.792	4.566	8.844
29	CTSE	cathepsin E	1510	1510	hCG18377.2	Hs.1355	3.38	2.602	0.778	2.742	9.454	7.318	8.844
30	SFTPB	surfactant, pulmonary-associated protein B	6439	6439	hCG34600.3	Hs.512690	3.377	2.588	0.79	2.742	9.139	11.346	8.844
31	PCMTD1	protein-L-isoaspartate (D-aspartate) O-methyltransferase domain containing 1	115294	115294	hCG27079.3	Hs.308480	3.351	2.577	0.775	2.742	8.844	1.684	8.844
32	EHMT2	euchromatic histone-lysine N-methyltransferase 2	10919	10919	hCG1811586.1	Hs.520038	-3.319	-2.96	0.359	3.656	11.424	-2.715	11.424
33	IL1B	interleukin 1, beta	3553	3553	hCG16263.2	Hs.126256	3.264	2.564	0.7	3.656	11.077	3.736	11.077
34	FOS	v-fos FBJ murine osteosarcoma viral oncogene homolog	2353	2353	hCG22355.3	Hs.568953|Hs.25647	3.254	2.553	0.7	4.569	13.44	4.863	11.424
35	CXCL3	chemokine (C-X-C motif) ligand 3	2921	2921	hCG16366.3	Hs.89690	3.25	2.542	0.708	4.569	13.056	6.875	11.424
36	MLLT4	myeloid/lymphoid or mixed-lineage leukemia (trithorax homolog, Drosophila); translocated to, 4	4301	4301	hCG2041523	Hs.448059	3.214	2.532	0.682	4.569	12.693	1.965	11.424
37	MGC17299	null	128218	128218	hCG1644000.4	Hs.104476	3.211	2.522	0.689	4.569	12.35	3.67	11.424
38	FLJ21125	null	79680	79680	hCG2002279.1	Hs.105642	3.199	2.511	0.687	4.569	12.025	1.612	11.424
39	MGC16943	null	112479	112479	hCG38246.3	Hs.248437	-3.195	-2.918	0.276	4.569	11.717	-2.408	11.717
40	SPINT2	serine peptidase inhibitor, Kunitz type, 2	10653	10653	hCG42856.3	Hs.31439	3.194	2.502	0.692	4.569	11.424	3.365	11.424
41	RDHE2	null	195814	195814	hCG1787435.2	Hs.170673	3.188	2.493	0.695	5.483	13.374	6.131	12.462
42	LOC401431	null	401431	401431	null	Hs.556097	-3.179	-2.885	0.295	5.483	13.056	-2.553	12.752
43	null	null	hCG1801741.2	null	hCG1801741.2	null	-3.156	-2.852	0.304	5.483	12.752	-2.66	12.752
44	AQP4	aquaporin 4	361	361	hCG37174.3	Hs.315369	3.147	2.484	0.663	5.483	12.462	8.626	12.462
45	BOC	null	91653	91653	hCG17534.2	Hs.556004	-3.129	-2.822	0.307	6.397	14.216	-1.776	13.056
46	CYP2B6	cytochrome P450, family 2, subfamily B, polypeptide 6	1555	1555	hCG1995522	Hs.1360	3.124	2.475	0.65	6.397	13.907	4.09	12.544
47	KIAA0703	null	9914	9914	hCG39788.3	Hs.6168	3.119	2.466	0.653	6.397	13.611	3.794	12.544
48	DSP	desmoplakin	1832	1832	hCG14677.2	Hs.519873	3.115	2.458	0.657	6.397	13.328	4.321	12.544
49	CYP4B1	cytochrome P450, family 4, subfamily B, polypeptide 1	1580	1580	hCG22100.5	Hs.436317	3.109	2.45	0.658	6.397	13.056	11.308	12.544
50	BAIAP2L1	BAI1-associated protein 2-like 1	55971	55971	hCG41326.4	Hs.567541	3.107	2.443	0.663	6.397	12.794	5.009	12.544
51	CXCL5	chemokine (C-X-C motif) ligand 5	6374	6374	hCG16364.3	Hs.89714	3.106	2.436	0.67	6.397	12.544	3.034	12.544
52	KIAA1799	null	84455	84455	hCG23452.4	Hs.269768	-3.1	-2.796	0.304	7.311	14.06	-1.769	13.056
53	SGK	serum/glucocorticoid regulated kinase	6446	6446	hCG32737.3	Hs.510078	3.083	2.428	0.655	7.311	13.795	2.573	13.293
54	null	null	hCG1643044.2	null	hCG1643044.2	Hs.128395|Hs.356460	-3.076	-2.773	0.303	7.311	13.539	-2.403	13.056
55	IL6	interleukin 6 (interferon, beta 2)	3569	3569	hCG38231.4	Hs.512234	3.066	2.42	0.646	7.311	13.293	6.385	13.293
56	VARSL	valyl-tRNA synthetase like	57176	57176	hCG1788592.2	Hs.102910	-3.065	-2.751	0.314	7.311	13.056	-2.06	13.056
57	TRIM23	tripartite motif-containing 23	373	373	hCG17860.4	Hs.567237	-3.062	-2.73	0.332	8.225	14.43	-1.608	14.06
58	null	null	hCG2037001	null	hCG2037001	null	3.056	2.413	0.643	8.225	14.181	3.682	13.484
59	OSM	oncostatin M	5008	5008	hCG41316.3	Hs.248156	3.042	2.406	0.637	8.225	13.941	2.487	13.484
60	null	null	hCG2041353	null	hCG2041353	null	3.037	2.399	0.638	8.225	13.708	1.964	13.484
61	SNF1LK	SNF1-like kinase	150094	150094	hCG401294.2	Hs.282113	3.037	2.392	0.644	8.225	13.484	3.292	13.484
62	OR6K3	olfactory receptor, family 6, subfamily K, member 3	391114	391114	hCG1645091.2	null	3.022	2.386	0.636	9.139	14.74	2.384	14.28
63	ATF3	activating transcription factor 3	467	467	hCG37734.3	Hs.460	3.018	2.38	0.639	9.139	14.506	3.171	14.28

64	RGS2	regulator of G-protein signalling 2, 24kDa	5997	5997	hCG41052.3	Hs.78944	3.017	2.372	0.645	9.139	14.28	3.436	14.28
65	NKD1	naked cuticle homolog 1 (Drosophila)	85407	85407	hCG24311.2	Hs.298434	-3.01	-2.711	0.3	9.139	14.06	-2.097	14.06
66	DENND3	DENN/MADD domain containing 3	22898	22898	hCG20740.4	Hs.18166	3.006	2.366	0.641	10.053	15.232	2.297	14.569
67	ABCC3	ATP-binding cassette, sub-family C (CFTR/MRP), member 3	8714	8714	hCG29634.3	Hs.463421	3.006	2.36	0.646	10.053	15.004	3.337	14.569
68	CLDN4	claudin 4	1364	1364	null	Hs.520942	2.998	2.354	0.644	10.053	14.784	2.277	14.569
69	null	null	hCG1820954.2	null	hCG1820954.2	null	2.983	2.348	0.634	10.053	14.569	2.771	14.569
70	TMC5	transmembrane channel-like 5	79838	79838	hCG38022.3	Hs.115838	2.976	2.342	0.634	10.967	15.667	4.127	15.446
71	FGFR4	fibroblast growth factor receptor 4	2264	2264	hCG1821375.1	Hs.165950	2.963	2.337	0.626	10.967	15.446	1.915	15.446
72	ST7L	suppression of tumorigenicity 7 like	54879	54879	hCG38462.3	Hs.201921	-2.962	-2.692	0.269	11.881	16.501	-2.032	15.429
73	C9orf58	chromosome 9 open reading frame 58	83543	83543	hCG30196.2	Hs.4944	-2.962	-2.675	0.286	11.881	16.275	-4.931	15.429
74	UROD	uroporphyrinogen decarboxylase	7389	7389	hCG17844.2	Hs.78601	-2.958	-2.658	0.3	11.881	16.055	-1.712	15.429
75	ARMC5	armadillo repeat containing 5	79798	79798	hCG1772019.3	Hs.121915	-2.954	-2.642	0.312	11.881	15.841	-1.694	15.429
76	null	null	ABI408610	null	null	null	2.95	2.331	0.618	11.881	15.632	1.918	15.632
77	ZBTB17	zinc finger and BTB domain containing 17	7709	7709	hCG25370.3	Hs.433764	-2.945	-2.628	0.317	11.881	15.429	-1.64	15.429
78	FLJ21839	null	60509	60509	hCG1990058	Hs.138207	-2.93	-2.614	0.316	12.794	16.403	-1.772	16.403
79	null	null	hCG1640549.4	null	hCG1640549.4	null	2.929	2.326	0.603	12.794	16.196	4.124	15.993
80	S100A9	S100 calcium binding protein A9 (calgranulin B)	6280	6280	hCG15465.3	Hs.112405	2.928	2.32	0.608	12.794	15.993	4.007	15.993
81	null	null	hCG1818462.1	null	hCG1818462.1	null	2.899	2.315	0.584	14.622	18.052	2.596	16.696
82	SFTPG	surfactant associated protein G	389376	389376	hCG2006673	Hs.211267	2.896	2.309	0.586	14.622	17.832	5.46	16.696
83	null	null	hCG1790588.2	null	hCG1790588.2	null	-2.896	-2.601	0.294	14.622	17.617	-2.321	16.858
84	LOC92270	null	92270	92270	hCG1641198.2	Hs.364045	-2.895	-2.588	0.307	14.622	17.407	-2.262	16.858
85	RGC32	null	28984	28984	hCG32189.3	Hs.507866	2.893	2.305	0.589	14.622	17.203	2.875	16.696
86	null	null	hCG2000660	null	hCG2000660	Hs.569727	-2.886	-2.575	0.31	14.622	17.003	-2.113	16.858
87	EIF1AY	eukaryotic translation initiation factor 1A, Y-linked	9086	9086	hCG37922.4	Hs.584171|Hs.461178	2.884	2.299	0.585	14.622	16.807	10.888	16.696
88	KPTN	kaptin (actin binding protein)	11133	11133	hCG20550.3	Hs.25441	-2.88	-2.563	0.317	15.536	17.655	-1.757	16.858
89	TOB1	transducer of ERBB2, 1	10140	10140	hCG1639758.3	Hs.531550	2.878	2.295	0.584	15.536	17.456	1.85	16.696
90	SKIV2L2	superkiller viralicidic activity 2-like 2 (S. cerevisiae)	23517	23517	hCG40991.3	Hs.274531	-2.877	-2.552	0.325	15.536	17.262	-1.554	16.858
91	null	null	hCG2042709	null	hCG2042709	Hs.530461	-2.874	-2.541	0.333	15.536	17.073	-2.217	16.858
92	DKFZp781N1041	null	387712	387712	null	Hs.372457	2.863	2.29	0.573	15.536	16.887	2.507	16.696
93	null	null	hCG1993095.1	null	hCG1993095.1	null	2.858	2.285	0.573	16.45	17.688	3.554	16.696
94	null	null	hCG2042092	null	hCG2042092	null	2.853	2.281	0.573	16.45	17.5	1.544	16.696
95	TUFM	Tu translation elongation factor, mitochondrial	7284	7284	hCG1747191.2	Hs.12084	-2.85	-2.529	0.321	16.45	17.316	-1.775	16.858
96	MAL2	mal, T-cell differentiation protein 2	114569	114569	hCG1653390.2	Hs.201083	2.848	2.276	0.572	17.364	18.087	5.943	16.696
97	BAI2	brain-specific angiogenesis inhibitor 2	576	576	hCG41602.3	Hs.524138	-2.847	-2.518	0.328	17.364	17.901	-3.149	16.858
98	LCP2	lymphocyte cytosolic protein 2 (SH2 domain containing leukocyte protein of 76kDa)	3937	3937	hCG37753.3	Hs.304475	2.846	2.271	0.575	17.364	17.718	2.654	16.696
99	ROS1	v-ros UR2 sarcoma virus oncogene homolog 1 (avian)	6098	6098	hCG1811669.2	Hs.1041	2.845	2.266	0.579	17.364	17.539	4.322	16.696
100	THBS4	thrombospondin 4	7060	7060	hCG2015975	Hs.211426	-2.843	-2.508	0.335	17.364	17.364	-7.001	16.858
101	UNQ5810	null	388218	388218	hCG1657150.4	Hs.115838	2.839	2.261	0.577	17.364	17.192	4.269	16.696
102	FLJ39660	null	284992	284992	hCG2043053.1	Hs.132519	-2.834	-2.499	0.335	17.364	17.023	-2.39	16.858
103	KIAA0372	KIAA0372	9652	9652	hCG1811113.2	Hs.482868	-2.828	-2.489	0.338	17.364	16.858	-1.509	16.858
104	GPRC5A	G protein-coupled receptor, family C, group 5, member A	9052	9052	hCG27693.3	Hs.194691	2.827	2.257	0.57	17.364	16.696	4.316	16.696
105	C14orf4	chromosome 14 open reading frame 4	64207	64207	hCG21245.3	Hs.179260	2.825	2.252	0.572	18.278	17.407	1.892	17.184
106	null	null	hCG2040471	null	hCG2040471	null	2.823	2.248	0.574	18.278	17.243	2.131	17.184
107	DKFZp686E2433	null	345462	345462	hCG2041603	Hs.445740	-2.811	-2.48	0.331	19.192	17.936	-1.718	17.135
108	CEBPE	CCAAT/enhancer binding protein (C/EBP), epsilon	1053	1053	hCG41750.2	Hs.558308	2.809	2.244	0.566	19.192	17.77	1.553	17.184
109	CAV2	caveolin 2	858	858	hCG39087.4	Hs.212332	2.808	2.239	0.569	19.192	17.607	2.324	17.184
110	PON2	paraoxonase 2	5445	5445	hCG1640820.2	Hs.530077	2.806	2.235	0.57	19.192	17.447	1.693	17.184
111	DDX3X	DEAD (Asp-Glu-Ala-Asp) box polypeptide 3, X-linked	1654	1654	hCG19318.3	Hs.380774	2.801	2.231	0.57	19.192	17.29	2.131	17.184
112	CDKL3	cyclin-dependent kinase-like 3	51265	51265	hCG1980962.1	Hs.105818	-2.8	-2.471	0.329	19.192	17.135	-1.922	17.135
113	ZNF300	zinc finger protein 300	91975	91975	hCG1980728	Hs.134885	-2.796	-2.462	0.333	20.106	17.793	-2.462	17.793
114	DUSP1	dual specificity phosphatase 1	1843	1843	hCG41245.2	Hs.171695	2.793	2.227	0.566	20.106	17.637	2.83	17.184
115	ABCA3	ATP-binding cassette, sub-family A (ABC1), member 3	21	21	hCG41022.5	Hs.26630	2.791	2.223	0.568	20.106	17.483	5.183	17.184
116	PEBP4	null	157310	157310	hCG15999.3	Hs.491242	2.79	2.219	0.571	20.106	17.332	4.073	17.184
117	null	null	hCG1998836.1	null	hCG1998836.1	null	2.788	2.215	0.573	20.106	17.184	1.506	17.184
118	TYRP1	tyrosinase-related protein 1	7306	7306	hCG27256.4	Hs.270279	2.781	2.211	0.571	21.02	17.813	3.868	17.663
119	LPP	LIM domain containing preferred translocation partner in lipoma	4026	4026	hCG17155.3	Hs.444362	2.772	2.207	0.565	21.02	17.663	1.718	17.663
120	ANKRD32	ankyrin repeat domain 32	84250	84250	hCG2016110	Hs.285055|Hs.482853	-2.769	-2.454	0.315	21.933	18.278	-1.849	17.832
121	null	null	hCG1817888.2	null	hCG1817888.2	Hs.498939	2.766	2.203	0.564	21.933	18.127	1.394	17.688
122	IDH3B	isocitrate dehydrogenase 3 (NAD+) beta	3420	3420	hCG39616.3	Hs.436405	-2.765	-2.446	0.32	21.933	17.978	-1.733	17.832
123	KIAA0649	KIAA0649	9858	9858	hCG27241.3	Hs.533260	-2.759	-2.438	0.321	21.933	17.832	-1.901	17.832
124	TREM1	triggering receptor expressed on myeloid cells 1	54210	54210	hCG32869.2	Hs.283022	2.758	2.199	0.559	21.933	17.688	6.164	17.688
125	NR4A3	nuclear receptor subfamily 4, group A, member 3	8013	8013	hCG28754.3	Hs.279522	2.749	2.196	0.553	22.847	18.278	4.115	17.711
126	ARF6	ADP-ribosylation factor 6	382	382	hCG2013574	Hs.525330	2.746	2.192	0.554	22.847	18.133	1.728	17.711
127	KIAA1772	KIAA1772	80000	80000	hCG1811231.5	Hs.54838	-2.745	-2.43	0.315	22.847	17.99	-1.862	17.849
128	ZNF228	zinc finger protein 228	7771	7771	hCG20223.4	Hs.48589	-2.744	-2.423	0.322	22.847	17.849	-1.639	17.849
129	CLDN18	claudin 18	51208	51208	hCG2022628	Hs.240182	2.743	2.189	0.555	22.847	17.711	6.311	17.711

130	IL17D	interleukin 17D	53342	53342	hCG31110.3	Hs.130652	-2.741	-2.415	0.326	23.761	18.278	-3.281	18.001
131	null	null	hCG2040182	null	hCG2040182	null	2.738	2.185	0.553	23.761	18.138	1.595	18.138
132	FAM40A	family with sequence similarity 40, member A	85369	85369	hCG40257.3	Hs.169577	-2.738	-2.409	0.329	23.761	18.001	-1.467	18.001
133	LOC92659	null	92659	92659	hCG1987587	Hs.336958	-2.73	-2.401	0.328	24.675	18.553	-2.335	18.543
134	FOSB	FBJ murine osteosarcoma viral oncogene homolog B	2354	2354	hCG20725.3	Hs.75678	2.726	2.182	0.544	24.675	18.414	2.514	18.414
135	null	null	hCG2041761	null	hCG2041761	null	2.716	2.178	0.538	25.589	18.955	2.015	18.815
136	RPS4Y1	ribosomal protein S4, Y-linked 1	6192	6192	hCG1988058	Hs.282376	2.715	2.174	0.54	25.589	18.815	46.164	18.815
137	null	null	hCG1640591.2	null	hCG1640591.2	null	-2.714	-2.394	0.32	25.589	18.678	-1.763	18.543
138	TGIF2	TGFB-induced factor 2 (TALE family homeobox)	60436	60436	hCG31789.3	Hs.292281	-2.714	-2.387	0.327	25.589	18.543	-1.725	18.543
139	BRD8	bromodomain containing 8	10902	10902	hCG39941.2	Hs.519337	-2.703	-2.38	0.323	26.503	19.067	-1.711	18.887
140	RBM35B	RNA binding motif protein 35B	80004	80004	hCG28119.2	Hs.436585	2.702	2.171	0.532	27.417	19.583	4.748	18.99
141	MMP7	matrix metallopeptidase 7 (matrilysin, uterine)	4316	4316	hCG1640914.2	Hs.2256	2.699	2.167	0.532	27.417	19.445	5.138	18.99
142	TACSTD2	tumor-associated calcium signal transducer 2	4070	4070	hCG22165.2	Hs.23582	2.696	2.164	0.532	27.417	19.308	4.867	18.99
143	TNFRSF10D	tumor necrosis factor receptor superfamily, member 10d, decoy with truncated death domain	8793	8793	hCG1645071.3	Hs.213467	2.692	2.16	0.532	27.417	19.173	2.835	18.99
144	ADCK4	aarF domain containing kinase 4	79934	79934	hCG1995823.1	Hs.130712	-2.691	-2.374	0.317	27.417	19.039	-1.509	18.887
145	SFTPA1	surfactant, pulmonary-associated protein A1	6435	6435	hCG2025128	Hs.465126	2.69	2.157	0.533	28.331	19.538	12.129	18.99
146	LOC284591	null	284591	284591	hCG2038553|hCG1995881	Hs.133183	-2.688	-2.368	0.321	28.331	19.405	-1.836	18.887
147	MGC33657	null	200373	200373	hCG1804625.3	null	2.688	2.154	0.534	28.331	19.273	1.537	18.99
148	SLC11A1	solute carrier family 11 (proton-coupled divalent metal ion transporters), member 1	6556	6556	hCG16301.2	Hs.471393	2.685	2.151	0.534	28.331	19.142	2.717	18.99
149	SSTR2	somatostatin receptor 2	6752	6752	hCG1641438.1	Hs.514451	-2.684	-2.361	0.323	28.331	19.014	-2.298	18.887
150	null	null	hCG1996962	null	hCG1996962	Hs.368518	-2.684	-2.355	0.328	28.331	18.887	-1.941	18.887
151	MYH9	myosin, heavy polypeptide 9, non-muscle	4627	4627	hCG41454.3	Hs.474751	2.682	2.147	0.535	29.245	19.367	2.033	18.99
152	BHLHB2	basic helix-loop-helix domain containing, class B, 2	8553	8553	hCG20358.2	Hs.171825	2.682	2.145	0.538	29.245	19.24	2.132	18.99
153	GATA6	GATA binding protein 6	2627	2627	hCG37191.2	Hs.514746	2.682	2.141	0.541	29.245	19.114	4.338	18.99
154	null	null	hCG1820902.1	null	hCG1820902.1	null	2.673	2.138	0.535	29.245	18.99	1.913	18.99
155	FLJ22688	null	80199	80199	hCG21121.3	Hs.288800	-2.666	-2.35	0.316	30.158	19.457	-1.903	19.457
156	C1orf116	chromosome 1 open reading frame 116	79098	79098	hCG1723946.2	Hs.32417	2.665	2.135	0.53	30.158	19.332	5.578	19.209
157	GLS	glutaminase	2744	2744	hCG25795.2	Hs.116448	2.664	2.132	0.532	30.158	19.209	1.739	19.209
158	ZFYVE1	zinc finger, FYVE domain containing 1	53349	53349	hCG21522.3	Hs.335106	-2.656	-2.343	0.313	31.072	19.666	-1.398	19.666
159	NAPSA	napsin A aspartic peptidase	9476	9476	hCG22926.2	Hs.512843	2.655	2.129	0.525	31.072	19.542	3.232	19.542
160	TTTY15	testis-specific transcript, Y-linked 15	64595	64595	hCG2040553|hCG2039064	Hs.433656	2.651	2.126	0.525	31.986	19.991	6.304	19.745
161	DDX20	DEAD (Asp-Glu-Ala-Asp) box polypeptide 20	11218	11218	hCG38167.3	Hs.485810	-2.649	-2.337	0.311	31.986	19.867	-1.735	19.867
162	NFKBIZ	nuclear factor of kappa light polypeptide gene enhancer in B-cells inhibitor, zeta	64332	64332	hCG38701.5	Hs.319171	2.648	2.124	0.524	31.986	19.745	2.464	19.745
163	AP2B1	adaptor-related protein complex 2, beta 1 subunit	163	163	hCG1992160	Hs.514819	-2.644	-2.331	0.313	32.9	20.184	-1.489	19.939
164	VPS16	vacuolar protein sorting 16 (yeast)	64601	64601	hCG2039972	Hs.269577	-2.643	-2.325	0.318	32.9	20.061	-1.523	19.939
165	CENPH	centromere protein H	64946	64946	hCG27171.3	Hs.200395	-2.639	-2.32	0.32	32.9	19.939	-2.103	19.939
166	KIFC3	kinesin family member C3	3801	3801	hCG1795607.2	Hs.23131	2.638	2.121	0.517	32.9	19.819	2.75	19.819
167	MAP3K5	mitogen-activated protein kinase kinase kinase 5	4217	4217	hCG33356.3	Hs.186486	2.637	2.118	0.519	33.814	20.248	2.292	20.248
168	null	null	hCG1650256.3	null	hCG1650256.3	null	-2.632	-2.314	0.318	33.814	20.127	-1.763	20.074
169	PPM1G	protein phosphatase 1G (formerly 2C), magnesium-dependent, gamma isoform	5496	5496	hCG21229.3	Hs.17883	-2.631	-2.309	0.322	34.728	20.549	-1.471	20.074
170	AMH	anti-Mullerian hormone	268	268	hCG2039529	Hs.112432	-2.63	-2.304	0.326	34.728	20.428	-2.64	20.074
171	PPT2	palmitoyl-protein thioesterase 2	9374	9374	hCG1999928.1	Hs.332138	-2.629	-2.298	0.331	34.728	20.309	-1.557	20.074
172	SYT11	synaptotagmin XI	23208	23208	hCG17193.3	Hs.32984	-2.629	-2.293	0.336	34.728	20.191	-2.212	20.074
173	FKSG14	null	64105	64105	hCG1747136.3	Hs.529778	-2.629	-2.289	0.34	34.728	20.074	-2.35	20.074
174	CD69	CD69 antigen (p60, early T-cell activation antigen)	969	969	hCG38142.3	Hs.208854	2.618	2.115	0.503	36.556	21.009	3.137	20.889
175	CYorf15B	chromosome Y open reading frame 15B	84663	84663	hCG1987333	Hs.145010	2.616	2.112	0.504	36.556	20.889	6.819	20.889
176	LNPEP	leucyl/cystinyl aminopeptidase	4012	4012	hCG1735557.1	Hs.554761	-2.606	-2.284	0.323	37.47	21.29	-1.561	21.206
177	ETV5	ets variant gene 5 (ets-related molecule)	2119	2119	null	Hs.43697	2.601	2.11	0.492	38.383	21.686	1.919	21.474
178	null	null	hCG2024913	null	hCG2024913	null	2.601	2.107	0.494	38.383	21.564	1.907	21.474
179	KIAA0664	KIAA0664	23277	23277	hCG32976.4	Hs.22616	-2.601	-2.279	0.322	38.383	21.443	-1.899	21.206
180	RAD54L	RAD54-like (S. cerevisiae)	8438	8438	hCG22090.2	Hs.523220	-2.6	-2.274	0.326	38.383	21.324	-2.275	21.206
181	MGC33648	null	133383	133383	hCG40616.3	Hs.85950	-2.599	-2.269	0.33	38.383	21.206	-2.11	21.206
182	ITPR1	inositol 1,4,5-triphosphate receptor, type 1	3708	3708	hCG21731.2	Hs.567295	2.59	2.104	0.486	39.297	21.592	2.164	21.474
183	CXCL1	chemokine (C-X-C motif) ligand 1 (melanoma growth stimulating activity, alpha)	2919	2919	hCG16368.2	Hs.789	2.59	2.101	0.489	39.297	21.474	2.206	21.474
184	CDH1	cadherin 1, type 1, E-cadherin (epithelial)	999	999	hCG28201.2	Hs.461086	2.584	2.099	0.485	40.211	21.854	6.424	21.854
185	STK11IP	serine/threonine kinase 11 interacting protein	114790	114790	hCG16070.3	Hs.22410	-2.58	-2.264	0.316	40.211	21.736	-1.48	21.736
186	LRRK2	leucine-rich repeat kinase 2	120892	120892	hCG1775001.3	Hs.187636	2.579	2.096	0.483	41.125	22.11	2.817	22.11
187	null	null	hCG2016784	null	hCG2016784	Hs.453331	2.569	2.093	0.476	42.039	22.481	2.285	22.481
188	THOP1	thimet oligopeptidase 1	7064	7064	hCG25247.3	Hs.78769	-2.565	-2.259	0.306	42.953	22.847	-1.981	22.726
189	PDZRN4	PDZ domain containing RING finger 4	29951	29951	hCG1997488	Hs.380044	-2.565	-2.255	0.31	42.953	22.726	-4.065	22.726
190	VIPR1	vasoactive intestinal peptide receptor 1	7433	7433	hCG28852.4	Hs.348500	2.564	2.091	0.474	42.953	22.607	3.992	22.607
191	ARHGAP8|LOC553158	Rho GTPase activating protein 8	23779	23779|553158	null	Hs.102336	2.559	2.088	0.471	43.867	22.967	3.66	22.612
192	HIST1H4A	histone 1, H4a	8359	8359	hCG1643898.2	Hs.248178	-2.557	-2.25	0.307	43.867	22.847	-2.151	22.847
193	BTG2	BTG family, member 2	7832	7832	hCG1640923.2	Hs.519162	2.556	2.085	0.471	43.867	22.729	2.426	22.612
194	DIP	null	23151	23151	hCG41796.3	Hs.475150	2.556	2.082	0.474	43.867	22.612	1.825	22.612
195	ANKRD13B	ankyrin repeat domain 13B	124930	124930	hCG29086.2	Hs.334715	-2.541	-2.246	0.295	46.609	23.902	-1.969	23.488

196	null	null	hCG1645220.1	null	hCG1645220.1	null	-2.538	-2.242	0.296	46.609	23.78	-2.52	23.488
197	ATRX	alpha thalassemia/mental retardation syndrome X-linked (RAD54 homolog, S. cerevisiae)	546	546	hCG20035.4	Hs.533526|Hs.571758	2.536	2.079	0.457	47.522	24.123	1.92	23.513
198	MIDN	midnolin	90007	90007	hCG21588.3	Hs.465529	2.536	2.077	0.459	47.522	24.001	1.825	23.513
199	LOC400506	null	400506	400506	hCG38020.2	Hs.300404	-2.535	-2.238	0.297	47.522	23.881	-1.642	23.488
200	LOC148413|LOC440746	null	440746	148413|440746	hCG20425.3	Hs.529943|Hs.515698	-2.534	-2.234	0.3	47.522	23.761	-1.532	23.488
201	STC1	stanniocalcin 1	6781	6781	hCG16685.3	Hs.25590	2.532	2.074	0.458	48.436	24.098	2.116	23.513
202	TIMM44	translocase of inner mitochondrial membrane 44 homolog (yeast)	10469	10469	hCG22555.3	Hs.465784	-2.529	-2.23	0.3	48.436	23.978	-1.55	23.488
203	null	null	hCG1994865	null	hCG1994865	Hs.533526	2.529	2.071	0.457	48.436	23.86	1.833	23.513
204	null	null	hCG1778862.2	null	hCG1778862.2	null	-2.527	-2.226	0.301	48.436	23.743	-1.688	23.488
205	LOC131873	null	131873	131873	null	Hs.477571	2.527	2.069	0.458	48.436	23.627	4.484	23.513
206	ZFP36	zinc finger protein 36, C3H type, homolog (mouse)	7538	7538	hCG43352.3	Hs.534052	2.526	2.066	0.459	48.436	23.513	2.709	23.513
207	FOXD1	forkhead box D1	2297	2297	hCG37843.4	Hs.519385	-2.521	-2.221	0.3	49.35	23.841	-3.298	23.488
208	null	null	hCG1774273.1	null	hCG1774273.1	null	-2.521	-2.217	0.304	50.264	24.165	-1.905	23.488
209	FLJ36032	null	284485	284485	hCG16929.3	Hs.297967	-2.52	-2.213	0.307	50.264	24.05	-1.892	23.488
210	JUNB	jun B proto-oncogene	3726	3726	hCG172481.3	Hs.25292	2.519	2.064	0.455	50.264	23.935	2.308	23.822
211	PERP	PERP, TP53 apoptosis effector	64065	64065	hCG17829.4	Hs.520421	2.519	2.061	0.458	50.264	23.822	2.395	23.822
